# Advanced Cellular Models for Rare Disease Study: Exploring Neural, Muscle and Skeletal Organoids

**DOI:** 10.3390/ijms25021014

**Published:** 2024-01-13

**Authors:** Cristina Bombieri, Andrea Corsi, Elisabetta Trabetti, Alessandra Ruggiero, Giulia Marchetto, Gaetano Vattemi, Maria Teresa Valenti, Donato Zipeto, Maria Grazia Romanelli

**Affiliations:** Department of Neurosciences, Biomedicine and Movement Sciences, University of Verona, 37134 Verona, Italy; cristina.bombieri@univr.it (C.B.); andrea.corsi@univr.it (A.C.); elisabetta.trabetti@univr.it (E.T.); alessandra.ruggiero@univr.it (A.R.); giulia.marchetto@univr.it (G.M.); gaetano.vattemi@univr.it (G.V.); mariateresa.valenti@univr.it (M.T.V.)

**Keywords:** organoids, iPSC, 3D cell culture, CRISPR/Cas9, rare diseases

## Abstract

Organoids are self-organized, three-dimensional structures derived from stem cells that can mimic the structure and physiology of human organs. Patient-specific induced pluripotent stem cells (iPSCs) and 3D organoid model systems allow cells to be analyzed in a controlled environment to simulate the characteristics of a given disease by modeling the underlying pathophysiology. The recent development of 3D cell models has offered the scientific community an exceptionally valuable tool in the study of rare diseases, overcoming the limited availability of biological samples and the limitations of animal models. This review provides an overview of iPSC models and genetic engineering techniques used to develop organoids. In particular, some of the models applied to the study of rare neuronal, muscular and skeletal diseases are described. Furthermore, the limitations and potential of developing new therapeutic approaches are discussed.

## 1. Introduction

Cell culture models are the most widely used in vitro techniques for basic research under controlled physiological conditions and for identifying new therapeutic targets for chronic or rare diseases. The availability of human cell models derived from patients with rare genotypes/phenotypes is pivotal for interpreting the pathogenic mechanisms of rare diseases. By studying cellular disease models, researchers may gain insights into how diseases develop, identify new molecular targets for specific therapies and test potential treatment approaches [1].

The main current strategies for studying the disease molecular mechanisms of the diseases are in vivo animal models, such as mouse, zebrafish and fruit fly, primary and secondary cell lines or/and engineered stem cells [2,3,4,5,6]. Although animal models can be useful for recapitulating complex organ structures and microenvironments and for studying organ function, species-specific differences hinder the translation of findings to humans. Data obtained in animal studies are often not reproducible in clinical studies, thus limiting the possibility of identifying therapeutic strategies. On the other hand, 2D cellular models from patient-derived specific cells cannot reproduce complex architectural structures or represent the interactions of the multicellular environment of the original organ. In addition, the limited proliferative potential of primary cell cultures may limit the type and number of feasible experiments [7,8,9]. Cell culture technology plays a crucial role in modeling the pathology of rare diseases and developing precision treatments. Although ideal, primary or patient-derived immortalized cells can be difficult to isolate or propagate indefinitely without avoiding immortalization, cell cycle deregulation and the risk that induced pluripotent stem cells (iPSCs) may lose their original phenotype. [10].

Patient-specific 3D organoid modeling systems represent in vitro cell models that can overcome some of these limitations. Organoids are three-dimensional structures derived from stem cells that closely resemble the structural and functional characteristics of specific organs or tissues of the body. These mini-organs are often created by growing stem cells in a specialized environment mimicking the natural environment in which an organ or tissue would normally develop [11]. Organoids can be created from different cell types, including embryonic stem cells (ESCs), iPSCs and adult stem cells (ASCs). The culture conditions employed can determine the specific cell types and structures formed within the organoid [11].

**Figure 1:**
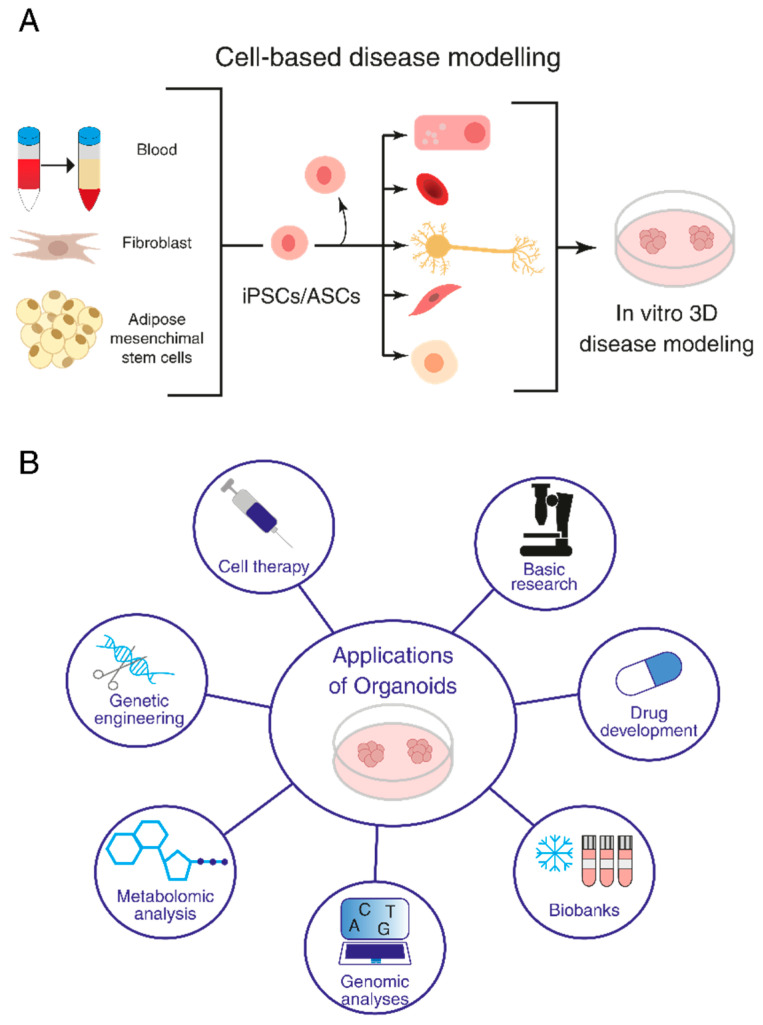
Organoids and their research applications. (**A**) Induced pluripotent stem cell (iPSC)- and adult stem cell (ASC)-derived organoids. (**B**) Application of organoids to basic research, investigation of disease mechanisms, drug screening and regenerative medicine.

Organoids have become a popular research tool in various fields, including developmental biology, regenerative medicine and disease modeling [12]. By replicating the morphology and functions of organs where cells grow in vivo and mimicking microenvironments and cell-to-cell and extracellular matrix interactions, organoids can provide insights into disease development and mechanisms, as well as serve as a platform for drug screening and personalized medicine (Figure 1).

The aim of this review is to provide a general overview of the technical aspects of organoid production with a focus on organoid models for rare neuronal, muscle and bone diseases. Section 1 describes general iPSC models, biomedical applications and genetic engineering techniques used to develop organoids, highlighting the differences, advantages and disadvantages of developing models based on iPSCs alone versus 3D organoid model systems, including the relevance of quality controls.

To provide an update on the application of iPSC-derived in vitro organoid techniques in the study of rare diseases, organoid models for rare neuronal, muscle and bone diseases are described. Without intending to be a comprehensive review of the literature on organoid disease models, the studies cited were selected on the basis of their heterogeneity, without focusing on a single model, but paying attention to the diversity of methodological approaches applied and results obtained. Finally, in Section 3, the main results derived from the literature selected for this review are summarized and commented on.

### 1.1. General Strategies Used for Organoid Production

Cell modeling involves culturing and studying cells in a controlled environment to mimic the characteristics of a particular tissue. This can be achieved by using various techniques, including genetic engineering, culturing cells in organ-on-chip and microfluidic technologies or introducing specific chemicals to induce disease-like conditions. Currently, organoids have been developed from various tissues and organs, including the brain, liver, intestine, kidney, pancreas and lungs [13]. They represent a promising tool for different fields of basic biological research, furthering our understanding of organ architecture and function [14,15]. Moreover, organoids make it possible to deepen our knowledge of embryonic development by studying cell–cell interactions and gene functions. Furthermore, 3D cultures have been applied to the study of human embryonic development and hematopoiesis through the study of the extra-embryonic lineage and three germ layers [16,17]. Once cells have been successfully modeled, researchers can use them to study disease mechanisms, identify potential drug targets and test the efficacy of potential treatments [18].

Several methods are applied to generate organoids [7], which differ depending on the tissue to be recapitulated [7]. Human brain organoids have been produced by pluripotent stem cells tightly compacted to promote their aggregation into embryonal bodies and then induced into neuroectodermal differentiation, embedded in an extracellular matrix and allowed to develop spontaneously in a spinning bioreactor [19]. Otherwise, organoids resembling organs of endodermic derivation, such as the lungs, intestine or stomach, require careful timing and selection of growth factors to modulate signaling pathways critical for proper organ development [7,20,21].

**Figure 2:**
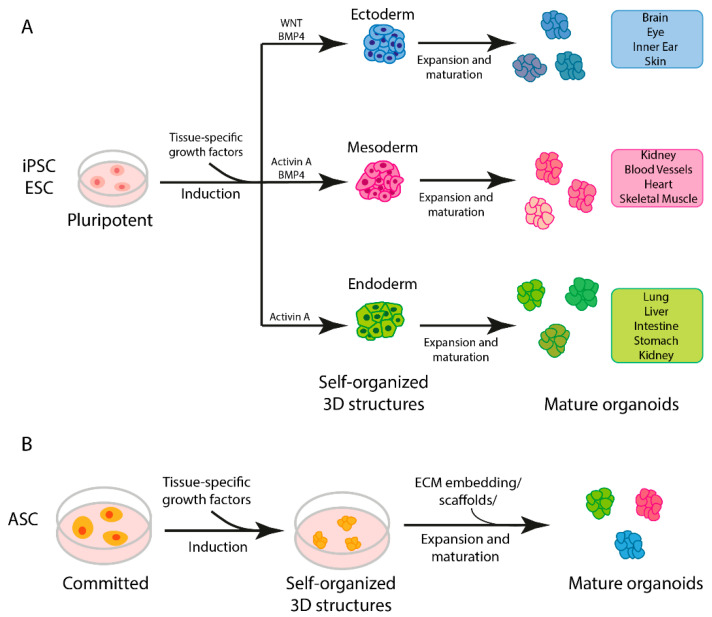
Steps in organoid generation. (**A**) Three-step generation of organoids from induced pluripotent stem cells (iPSCs) and embryonic stem cells (ESCs). (**B**) General protocol for generation of adult stem cell (ASC)-derived organoids.

Taking these differences into account, however, it is possible to identify a general three-step procedure applied in the protocols currently used to generate organoids (Figure 2A) [7]. Starting with pluripotent stem cells (iPSCs or ESCs), the first step requires differentiation toward a specific embryonic germ layer of interest (ectoderm, mesoderm or endoderm) using selected factors that activate cell differentiation commitment, such as WNT, BMP4 and activin A [7]. In the next step, the cells are differentiated into the target tissue/organ by the addition of tissue-specific growth factors. Finally, the cells are embedded in an ECM gel or aggregated in a 3D structure (using scaffold-forming external biomaterials [22]) to expand [7]. If, instead, ASCs are used as the starting material, the first step of germ-layer differentiation is not necessary, as this kind of stem cell already possesses some degree of commitment (Figure 2B) [7].

**Figure 3:**
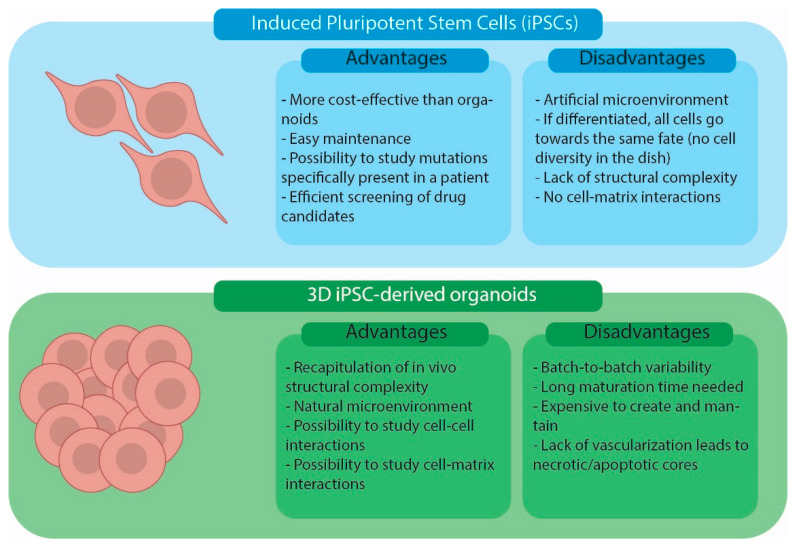
Advantages and disadvantages of iPSC cell cultures and iPSC-derived organoid models.

Two-dimensional iPSC cultures and iPSC-derived organoids are in vitro cellular models currently used in biomedical research. The advantages and disadvantages of using the two models are summarized in Figure 3. Simple iPSC monolayer cultures offer a cost-effective and easily manageable way to model a disease using patient cells [9]. Moreover, they can be used as an economical and quick way to assess the effectiveness and adverse effects of drug candidates [23]. Two-dimensional cultures are a good model for epithelial tissues that better mimic the cell-matrix interaction in vivo [24,25]. For non-epithelial tissues, 2D iPSC cultures present some limitations due to a lack of cell-to-cell and cell-to-ECM interactions [6,9,26]. Three-dimensional iPSC-derived organoids, instead, offer the possibility of studying cells in a more natural environment where cell-to-cell and cell-to-ECM interactions are present and the overall system architecture resembles that present in human organ/tissues [6,7,9,26]. Nevertheless, current human organoid models have limitations. Indeed, they are expensive to generate and maintain, require long maturation periods and lack proper vascularization, resulting in the necrosis and apoptosis of some cells [9]. Finally, organoids tend to show a large degree of variation from batch to batch, limiting the reproducibility of the technique [9,27,28]. 

### 1.2. Biomedical Applications

Organoids generated either from iPSCs or tissue biopsies of patients containing ASCs may be applied in clinical research for modeling infectious, hereditary and tumor diseases [29]. 

In the study of infectious diseases, the application of organoids sheds light on viral pathogenesis from virus infection to replication, mimicking virus–host interactions. Recent studies have highlighted the potential application of liver organoids for Hepatitis E virus infection (HEV), as this model system, unlike 2D cell cultures, maintains the polarity and homeostasis necessary for viral infection and reproduction, supporting their innovative use in discovering new anti-HEV drugs [30]. An organoid model was also useful in clarifying the interaction between human parasite microorganisms and epithelium targets, revealing important consequent transcriptomic changes relating to the life cycle of *Cryptosporidium* [31]. Moreover, neural organoids allowed for the identification of potential therapeutic compounds to treat Zika virus (ZIKV) infection [7,32,33]. 

A 3D culture has been applied to study human embryonic development and hematopoiesis through the study of the extra-embryonic lineage and three germ layers [16]. Furthermore, human retinal organoids and their single-cell RNA-seq analysis highlighted, in great detail, the development of the retina [17].

Organoids isolated from tissues from patients may also be suitable for biobanking as a resource for genotype/phenotype correlation analyses, the study of rare mutations causing diseases, the correction of genetic defects and regenerative medicine, leading to the development of future precision medicine applications. In addition, recent genome editing technologies applied to organoids allow for the study of some defects in a controlled microenvironment, confirming the valuable use of these systems for basic research and therapeutic development [34]. 

Human organoids have also been developed from several different cancer cells, isolated from both primary tumors and metastasis. Many genetic analyses have revealed that these “tumoroids” highly conserve their genomic landscape, making them a suitable source to investigate genotype–phenotype correlations and perform drug screening, confirming the importance of organoids in terms of biobanking and the subsequent development of precision medicine [35]. Gut organoid models are among the most developed and have been intensively described in recent reviews [36,37,38,39]. Colon organoids represent useful applications for the identification of potential drug targets in the treatment of genetic disorders. For instance, human colonic organoids have been developed as a phenotypic screening tool for drug selection in therapeutic interventions for cystic fibrosis (CF). Intestinal epithelium organoids have been a valuable tool to monitor chloride-channel function using forskolin-induced swelling assays, allowing for the selection of efficacious treatments for patients with rare CFTR mutations [40]. The development of human brain organoids is also contributing to shed new light on neuronal diseases and screening to design personalized therapies, such as for Autism Spectrum Disorders and Alzheimer’s and Parkinson’s diseases [41].

### 1.3. Cutting-Edge Technologies for Genetic Modifications of iPSCs and Organoids

The development of iPSCs and their differentiation through the formation of organoids that morphologically and physiologically resemble human tissues already represent significant progress, as described above. However, in some experimental settings, further genetic modification of iPSCs and organoids may be required before downstream applications to address basic biological and biomedical questions [42]. Advances in genome editing technologies make it possible to precisely edit specific genes in iPSCs, modifying them to express or induce specific characteristics. The editing of iPSCs may be necessary to induce differentiation towards specific cell types, modify genetic defects or modulate specific pathways. This section will provide an overview of the available state-of-the-art editing approaches and their applications in iPSCs and organoids.

To introduce gene-editing components into iPSCs and organoids, several approaches exist, depending on the properties of the target cells, the size of the DNA fragment and the required duration of gene expression [13]. For example, the use of retroviral or lentiviral vectors may be considered if the modified gene does not exceed 8 kb and needs to be stably integrated into the host genome of non-actively replicating cells. On the other hand, adenoviruses allow episomal expression in target cells and permit the insertion of large DNA fragments (>8 kb) in non-dividing cells such as brain cells/organoids [43,44,45,46]. Other non-viral methods, such as electroporation or the lipofection of naked DNA, are available, but it must be considered that transgene expression is transient and lipofection may affect cell survival.

Developing zinc finger nucleases (ZFNs) by assembling zinc finger DNA-binding domains with the cleavage domain of the restriction endonuclease FokI represents one of the first successes in the construction of programmable artificial endonucleases. The search for other endonucleases that can be more easily reprogrammed continued, leading to the identification of a family of transcription activator-like effector (TALE) proteins in plant pathogens. Their modular structure for DNA recognition can be used to construct ad hoc TALE nucleases (TALENs) by appropriately reassembling TALE domains, similar to what has been described for zinc finger domains.

A huge step forward in genome editing was made with the discovery of CRISPR/Cas9, a natural system used by several prokaryotes for adaptive molecular immunity against bacteriophage infections. Its mechanism was subsequently modified and engineered for use in eukaryotic cells to allow for the cutting or editing of specific DNA sequences in a target cell. Specifically, the CRISPR/Cas9 system consists of two components, the Cas9 endonuclease and a single-guided RNA (sgRNA or gRNA), which directs the Cas9 nuclease to a specific sequence, where it introduces a double-strand break (DSB). After cleavage by the Cas9 nuclease, double-strand breaks can be repaired either by homology-directed repair (HDR), a precise and high-fidelity repair system, or by non-homologous end joining (NHEJ), in which the blunt ends are bound together. Repair by HDR, through recombination with a suitably supplied homologous DNA sequence, allows researchers to introduce sequence-specific changes in the target gene, while repair by NHEJ alters the reading frame and thus the translation of the target gene.

In general, mutagenesis can take place either in a tissue that serves as a source of adult stem cells or in isolated cells used to generate organoids; the second option is preferable as it is more efficient and less costly. Over the years, attempts have been made to genetically modify different types of ASC-derived organoids, as described in detail by others [13,42]. Schwank et al., Matano et al. and Kavasaki et al. are just some of the researchers showing that CRISPR/Cas9 could be applied in gastrointestinal tract organoids for gene knock-out or mutation repair [47,48,49]. CRISPR/Cas9 was also used on liver organoids in a study in which a retroviral vector was used to deliver modified genes [35]. Furthermore, Dekkers and colleagues showed that by using CRISPR/Cas9 editing in mammary epithelial organoids, it is possible to model clonal evolution in breast cancer development [50]. Further efforts have been made to induce gene editing in brain organoids to decipher disease mechanisms and development, as reported more specifically in the following sections dedicated to rare neurological disorders and cancers. CRISPR/Cas9-mediated gene editing is usually conducted on brain organoid founder cells, i.e., ESCs and iPSCs [45,51,52,53,54,55]. 

CRISPR/Cas9 is also widely used to study congenital nervous system malformations such as microcephaly. Bendriem et al. used genome editing to knock out the gene Occludin (OCLN) in mouse and human models. OCLN KO resulted in early neuronal differentiation disorder, the slow self-renewal of progenitor cells and increased apoptosis in mice, whereas the human neural progenitor cells were seriously affected [56,57,58]. Leigh syndrome (LS) is another hereditary progressive neurodegenerative disease that leads to subacute necrotizing encephalomyelitis [56,57]. Cell models of LS have been generated by using iPSCs from LS patients with a mutation in the gene for surfeit locus protein 1 (SURF1) to produce brain organoids. The combined approach of SURF1 gene editing by CRISPR-Cas9 and iPSCs successfully restored a normal morphology in an organoid culture [57].

Genome editing is still a growing research field, and new methodologies are being developed for organoids and iPSCs. Nonetheless, as shown by the studies reported in this section, the combined application of CRISPR/Cas9 and organoid models may provide a technical platform for studying organ development, rare diseases and congenital malformations.

### 1.4. Quality Controls

Genetic manipulation in organoids requires further standardization in both the choice of strategies to introduce the modified gene and the most suitable methodology for genome editing [13]. An important aspect that needs further discussion is the design of proper controls to verify that genetic manipulation occurs only in the target regions. CRISPR/Cas9 appears to be more precise and accurate than programmable endonucleases based on ZFN or TALEN; however, the risk of “off-target” events that modify unwanted genome regions is still present. Some quality controls are available to verify the absence of off-target effects. One approach is to bioinformatically predict possible off-target regions and consequentially sequence the region to verify the absence of undesired editing [59,60]. Another considerably more expensive but accurate method is to use whole-genome sequencing to check for alterations in a cellular genome.

Extensive quality controls are required to validate data from iPSC-derived organoids due to donor genetic variability, laboratory techniques and cell differentiation differences. The general standards for data quality derived from organoid studies are based on validating organoid composition and structure [12]. The constant monitoring of organoids’ structural and cellular characterization requires live organoid imaging, time-lapse imaging of morphological changes and immunostaining [61,62,63]. Standardization on the dissociation, passage and cryopreservation of organoids is also required. Particular attention needs to be dedicated to the control of long-term organoid cultures due to the development of a necrotic core in static cell cultures, a loss of cells during culture medium changes and a lack of oxygen and nutrients in the core of organoids [64,65]. Despite the refinement of validation methods, the heterogeneity and reproducibility, both morphological and functional, of the obtained 3D organoid systems remain the most critical aspects when transferring the results to preclinical models.

## 2. Organoids and iPSC-Based Models for Rare Neurodegenerative, Neuromuscular and Skeletal Diseases

Cellular models, such as iPSCs and 3D organoids, generated either from iPSCs or from patient tissue biopsies, are valuable models for studying rare diseases, including the role played by causal mutations in disease development and progression. Table 1 lists representative rare neurodegenerative and neuromuscular diseases, skeletal disorders and brain tumors for which iPSC/organoid models have been generated. These examples of cellular model applications will be described in the following sections. 

### 2.1. Modeling Rare Neurodegenerative Diseases and Tauopathies with iPSC-Derived Organoids

The nervous system is a highly complex structure composed mainly of neurons and glial cells organized in a complex tissue architecture. Many characteristics, such as morphological features and specific cell populations, particularly for the human brain, are species-specific [106], thus limiting the information obtained from studies using classical cell cultures and animal models. This began the quest for more complex and physiologically relevant in vitro models that could to mimic the basic structure and cell content of the human nervous system. Some of these complex organoid systems have been used to model rare neurological diseases, as described below.

#### 2.1.1. Autosomal Recessive Primary Microcephaly (MCPH3)

Cerebral organoids (COs), used to model several rare neurological diseases, are 3D self-organizing cultures of cerebral brain tissue differentiated from pluripotent stem cells, including iPSCs, which include different neural and neuronal subtypes spatially organized in a manner similar to a developing human brain and closely recapitulating developmental gene expression patterns [22,52]. Pioneering work on cerebral organoids began with the human iPSC-derived cerebral organoid implemented by Lancaster et al. in 2013 [19]. This model developed interdependent brain regions, including the cerebral cortex, which contains progenitor populations that organize and produce mature cortical neuron subtypes. Cerebral cortical areas in the organoid showed an organization similar to a developing human brain in the early stages, with the presence of a considerable population of outer radial glial stem cells. Moreover, in this study, microcephaly patient-derived iPSCs and shRNA were used to develop an organoid model for the investigation of the role of CDK5 regulatory subunit-associated protein 2 (CDK5RAP2). Microcephaly patient organoids demonstrated premature neuronal differentiation, a defect that could explain the disease phenotype [19].

#### 2.1.2. Miller–Dieker Syndrome (MDS)

Miller–Dieker syndrome (MDS) is the most severe form of lissencephaly, a genetic neurological disorder associated with mental retardation and intractable epilepsy [45]. Classical lissencephaly is primarily due to heterozygous mutations or deletions of the LIS1 gene, which encodes a protein that is part of the LIS1/NDEL1/14,3,3e complex and essential for dynein regulation and microtubule dynamics [107]. MDS is caused by a heterozygous deletion of chromosome 17p13.3 involving two of these genes, LIS1 and the tyrosine 3-monooxygenase/tryptophan 5-monooxygenase activation protein epsilon (YWHAE) coding for 14.3.3ε. This deletion leads to very severe malformations with neuronal disorganization during cortical development [52]. An analysis of forebrain-type organoids derived from MDS-specific iPSCs revealed that a deficiency of the LIS1/NDEL1/14.3.3ε complex leads to an impairment of cortical niche signaling, resulting in alterations in N-cadherin/b-catenin signaling and a non-cell-autonomous expansion defect of ventricular zone radial glia cells. These patient-derived organoids were indeed significantly smaller, with an asymmetric cell division, alterations in microtubule organization and a disruption of the cortical niche architecture. The CRISPR/Cas9 genome editing applied to repair the two defective genes in MDS forebrain organoids contributed to analyzing the level of malformations during cortical development following the insertion of the genes [52].

In another study, an iPSC-derived cerebral organoid model of early corticogenesis was implemented to investigate the effects of MDS mutations on human progenitor subtypes that control neuronal output and influence brain topology in the first trimester of cortical development. Cerebral organoids derived from control and MDS iPSCs were analyzed using multiple approaches, including live-cell imaging, immunostaining and single-cell RNA sequencing. The results show cell-type-specific defects of lissencephaly across neuroepithelial cell expansion, abnormalities in neuronal migration and a mitotic defect of outer radial glia progenitors, a cell type significant for human cortical development but largely absent from lissencephalic rodents. Furthermore, the correction of the MDS causative chromosomal deletion corrected the cell migration defect. These findings support the hypothesis that outer radial glia dysfunction may be a feature of cortical malformations associated with lissencephaly [45].

#### 2.1.3. Leukoencephalopathy with Vanishing White Matter (VWM)

Leukoencephalopathy with vanishing white matter (VWM) is a rare autosomal recessive disease characterized by variable neurologic features associated with white matter lesions in brain imaging. This neurological disorder can be caused by mutations in any of the EIF2B1-5 genes (VWM 1-5) which encode subunits of eukaryotic translation initiation factor 2B (eIF2B) [108]. Alterations in this factor influence embryonic brain development, as demonstrated by the neuropathological characteristics of two fetuses carrying EIF2B5 gene mutations [109]. To investigate the pathophysiological mechanisms in the dynamic process of VWM development, wild-type and eIF2B mutant brain organoids were developed from iPSCs [66]. The mutant organoids showed a smaller size in the early stages, with increased apoptosis due to the overactivation of the unfolded protein response. This finding can explain the presence of microcephaly in patients with the congenital form of VWM. The results of this study suggest that mutations in eIF2B genes cause a delay in the development of neural stem cells of the brain organoids, which in turn affects subsequent glial cell development [66].

#### 2.1.4. Huntington Disease (HD)

Both human iPSCs and brain telencephalic organoids have been developed to evaluate the neuronal defects associated with Huntington’s disease (HD), a neurodegenerative autosomal dominant disease with a late onset caused by a CAG expansion in the huntingtin (HTT) gene. HD symptoms include debilitating chorea, cognitive impairment and psychiatric impairment and are mainly related to neuronal loss in the striatum and cortical areas. Mutant HD organoids exhibit a high rate of immature cells and an abnormal acquisition of mature neuronal markers [110]. Both 2D and 3D lines harboring mutant HTT mature more slowly than lines unaffected by the disease or show altered gene expression in early developmental stages in both striatal and cortical regions [67]. The morphological features of the cells and the tissue organizational characteristics of 3D organoids, such as a disrupted spatial cytoarchitecture, deficient cellular compartmentalization and similarities to the immature ventricular and subventricular zones seem to indicate that mutant HTT plays a pivotal role in slowing fetal neurodevelopment [111]. Gene expression studies performed in both 2D and 3D cultured systems carrying HD mutations have identified altered expression of genes involved in sodium voltage-gated currents (SCN1A, SCN2A, SCN4B, SCn9A), neuronal migration and differentiation (SOX11, GAP43, CELSR3) [67,110,112]. These findings agree with research suggesting a critical role of HTT in neurogenesis and embryonic tissue organization in early development. In addition, they may explain neurodevelopmental disorders, along with the more specific neurodegenerative ones, shown by the juvenile form of HD caused by the largest CAG expansions. [67,110].

#### 2.1.5. Creutzfeldt–Jakob Disease (CJD)

Cerebral organoids have been proposed as valuable models for investigating human prion diseases and their subtypes. These 3D models can be infected with different subtypes of Creutzfeldt–Jakob disease (CJD) prions, which in humans cause different manifestations of the disease, maintaining the original subtype characteristics of the infecting prions. Mouse models expressing human prion proteins inoculated either with cerebral organoids that had been infected with two CJD subtypes (MV1 and MV2) or with the original human brain material present similar disease characteristics, demonstrating that this in vitro model can faithfully reproduce different subtypes of prion disease [68,113]. 

#### 2.1.6. Retinitis Pigmentosa (RP)

Retinitis pigmentosa (RP) is a group of heterogeneous inherited retinal degenerative diseases characterized by the irreversible loss of photoreceptor cells, resulting in early-onset progressive visual field defects and irreversible blindness. Several different genes can be involved in RP development, leading to retina-specific phenotypes [69,70,71]. Retinal organoids (RO) and retinal pigment epithelium (RPE) cells were derived from the iPSCs of patients carrying different RP gene mutations, providing information about their pathogenesis and allowing for disease modeling, a comparison of the disease mechanisms and drug testing on the different genetic forms of RP. Patient-specific organoids have been differentiated from iPSCs derived from RP patients with different mutations in the following genes: the retinitis pigmentosa GTPase regulator (RPGR) gene, the most common cause of this disease; the beta subunit of the rod cGMP-phosphodiesterase type 6 (PDE6B) gene [69]; the usherin (USH2A) gene, one of the most common causes of non-syndromic RP [70]; the RHO gene [74]; and the pre-mRNA processing factor 31 (PRPF31) gene, which causes one of the most common forms of dominant RP [75]. Each one of these models presents important morphological and functional changes mimicking the typical human phenotype of the respective RP form, also allowing the different gene mutations to be associated with significant defects in photoreceptors in terms of morphology, localization, transcriptional profiling and electrophysiological activity. 

Significant efforts are directed toward developing new therapeutic strategies, implementing RO-based cell therapy and providing patient-derived ROs for gene therapy, thus promoting the development of personalized medicine [71]. The CRISPR-Cas9-mediated correction of the RPGR mutation restored gene expression and rescued the photoreceptor’s structure and electrophysiological properties [72,73]. Treatment with PR3, a small molecule targeted to Nuclear Receptor Subfamily 2 Group E Member 3 (NR2E3), attenuates the increased Rhodopsin (RHO) expression and partially rescues the altered RHO trafficking in an iPSC-derived retinal organoid implemented from a patient with a copy number variation in the RHO gene [74]. The defective RPE phenotype and photoreceptor cell death in RO linked to the presence of a PRPF31 mutation can be prevented by PRPF31 gene supplementation using the CRISPR/Cas9 strategy. The restoration of PRPF31 expression can reverse the photoreceptor’s defective phenotypes and rescue the mutation-induced photoreceptor cell death, paving the way for a potential AAV-mediated gene therapy for the treatment of PRPF31-related RP [75].

#### 2.1.7. Charcot–Marie–Tooth (CMT) Disease

Charcot-Marie-Tooth (CMT) disease is an inherited neuropathy that is clinically heterogeneous and associated with mutations in a wide spectrum of genes that mainly affect the peripheral nervous system (PNS) [114]. According to the primary deficit site, CMT is classified into demyelinating (CMT1) forms, affecting Schwann cells, and axonal (CMT2) forms, occurring in neuronal axons. CMT1A is caused by a tandem duplication on chromosome 17p11.2-p.12 containing the Peripheral Myelin Protein 22 (PMP22) gene, which specifically affects the myelination of peripheral nerves [114]. The increased level of PMP22 expression derived from this tandem duplication disturbs the myelination process. A complex organoid culture modeling the cell content and basic structure of the human PNS was developed by Van Lent and colleagues [76] from patient-derived CMT1A iPSCs. This organoid system, composed of multiple self-organizing and interacting cell types, including myelinating Schwann cells, recapitulates many of the early disease hallmarks. The downregulation of PMP22 expression using short-hairpin RNAs or a combination of drugs was able to ameliorate myelin defects in CMT1A organoids, which also supports the role of PMP22 expression inhibitors as a promising therapeutic approach for this disorder. This study paves the way for the use of iPSC-derived organoids containing myelinating Schwann cells to potentially study other demyelinating neuropathies of the PNS, such as Guillain–Barré syndrome, chronic inflammatory demyelinating polyneuropathy or schwannomatosis. Moreover, Van Lent et al. hypothesize that organoids infected with Mycobacterium leprae, which is the cause of leprosy, can be used to investigate the invasion of these bacteria in Schwann cells [76]. Van Lent and colleagues have also generated a patient-derived iPSC model to identify common traits of axonal degeneration shared by different subtypes of Charcot–Marie–Tooth Type 2 (CMT2) [77]. This study compared the cellular phenotypes of iPSC-derived neurons from patients affected by different CMT2 subtypes, covering the most frequent CMT2-causing genes, and healthy controls. Furthermore, it was demonstrated that the developed iPSC-derived neurons are true hallmarks of CMT2A since the CRISPR/Cas9 correction of the pathogenic Mitofusin 2 MFN2-R94Q patient iPSC line prevents the characteristic disease phenotypes. CMT2-derived motor neurons showed neuritic network deficits with extracellular electrophysiological alterations, as well as progressive deficits in mitochondrial and lysosomal trafficking. A common mitochondrial dysfunction in CMT2-derived motor neurons, with abnormalities in morphology, expression pattern and oxidative phosphorylation, was observed across the different CMT2 subtypes. Moreover, the inhibition of a dual leucine zipper kinase partially ameliorates the mitochondrial dysfunction in the CMT2A and CMT2E subtypes [77].

#### 2.1.8. Frontotemporal Dementia (FTD)

Human iPSC-derived brain organoids, being a valuable model to study 3D multicellular interaction occurring in cortical tissue [115,116,117,118], have been successfully applied to study genetic mutations in frontotemporal dementia [80]. An advanced model of human cortical organoids has been developed for studying the early molecular steps that occur in the preclinical phase of amyotrophic lateral sclerosis overlapping with frontotemporal dementia (ALS/FTD), an untreatable neurodegenerative disease characterized by rapid cognitive decline and paralysis [119]. The researchers exploited brain organoid slicing methods that improve the viability of the cell composition in the growing cultures. Specifically, they cultured human cortical organoids from iPSCs derived from patients, carrying the hexanucleotide repeat expansion mutation C9ORF72, and demonstrated, using hundreds of organoid slices, specific transcriptional, proteostasis and DNA repair alterations in astroglia and neurons. 

An iPSC-derived 3D co-culture model consisting of mature-like neurons and astrocytes was used to study the pathology of a TAR DNA-binding protein (TDP-43) in ALS and FTD. In this model, the depletion of granulin (GRN) expression mimics the features of the TDP-43 proteinopathy, represented by an extranuclear accumulation of hyperphosphorylated TDP-43 and an altered splicing of Stathmin 2 (STMN2), a protein involved in neuronal survival [81]. FTD is included in the spectrum of neurodegenerative *tauopathies* [120]. The impact of different mutations in the MAPT gene encoding for the Tau protein was studied in cerebral organoids derived from patients with FTD, demonstrating significant differences in the expression of several genes, including the ceramide synthetase genes CERS4, CERS5 and CERS6, and PINI, TBK1, FUS and ELAV4 [78,121]. In particular, using human cerebral organoids, it was shown that the MAPTp.V3337M mutation induces Tau phosphorylation, glutaminergic dysfunction and a loss of glutamatergic neurons [78]. Furthermore, in organoid astrocytes carrying the Tau V337M and R406W mutations, the upregulation of several genes involved in the cholesterol biosynthesis pathway, including HMGCR, ACAT2, STARD4, LDLR and SREB2, was found [79]. Dissociated organoids were used to study the genotype–phenotype relationship using iPSCs from patients carrying the MAPTp.R406W mutation. In this model, patient-derived neurons showed morphological and functional abnormalities that were rescued by microtubule stabilization [122]. Furthermore, the role of p25/Cdk5 in frontotemporal dementia was demonstrated by patient-derived iPSCs carrying the Tau P301L mutation and generating P301L:Δp35KI isogenic iPSC lines using CRISPR/Cas9 genome editing. Cerebral organoids from isogenic iPSCs demonstrate the crucial role of p25/Cdk5 in mediating Tau-associated pathology [123].

### 2.2. hiPSCs, Organoids and Novel Platforms for Neuromuscular Disorder Modeling

Skeletal muscle is a peculiar tissue characterized by an elaborate architecture of multinucleate contractile myofibers together with motor neurons, endothelial and immune cells, perivascular and connective tissue and muscle stem cells (MuSCs) [124]. The maintenance of this complex environment ensures the normal function, homeostasis and regeneration of muscle tissue [124]. The gold standard for studying skeletal muscle dynamics in health and disease has long been represented by well-established 2D cell cultures and animal models, mostly mice. Despite their undeniable utility, their use is limited by inherent issues that cannot be overcome.

Thanks to extraordinary advances in knowledge and technology, promising new models for various skeletal muscle diseases have been developed in recent years. From iPSCs to organoids and organ-on-a-chip, several strategies are emerging to investigate neuromuscular disorders [125]. Human iPSCs can be obtained from different somatic cell sources, including fibroblasts, peripheral blood mononuclear cells and myoblasts [126]. Different protocols [124,127,128] are now available to induce iPSC-derived myogenic progenitor cells in vitro through the transgenic overexpression of key myogenic transcription factors or directly using cocktails of signaling molecules and growth/inhibition factors [124,125]. These iPSCs can be a powerful 2D culture platform alone or in co-culture with inflammatory, neuronal and endothelial cells [125,129,130,131]. Even more innovatively, iPSCs represent the starting material for building 3D engineered skeletal muscle tissue using natural- or synthetic-scaffold-based models, 3D bioprinting technology or organ-on-a-chip platforms [125,129,130,131]. The great potential of these tools lies in the possibility of achieving endless proliferative capacity and controllable differentiation from patients’ iPSCs up to the production of functional artificial muscles. They may recapitulate the complex muscle tissue architecture and microenvironment, as well as molecular, biomechanical and electrophysiological dynamics. 

Human iPSC-derived muscle fibers have recently been utilized in monolayer cultures to model several skeletal muscle disorders, such as facioscapulohumeral dystrophy (FSHD) [132,133], Duchenne muscular dystrophy (DMD) [134,135,136,137], limb–girdle muscular dystrophies (LGMDs) [93,138,139,140], myotonic dystrophy type 1 (DM1) [141,142], LAMIN A/C (LMNA)-related muscular dystrophies [143], infantile Pompe disease [144,145], McArdle disease [146], carnitine palmitoyltransferase II (CPT II) deficiency [147] and nemaline myopathy [148], allowing for a good replication of the main disease-related pathological features.

A scaffold-based 3D approach has recently been applied to model genetic muscle diseases. Human iPSCs from patients with different types of muscular dystrophies, including Duchenne muscular dystrophy (DMD), limb–girdle muscular dystrophy type R3 (LGMDR3) and LMNA-related muscular dystrophies, were used to successfully generate artificial muscles that recapitulate the contractile deficit of DMD associated with a reduced expression of fast myosin isoforms in DMD mutant iPSC-derived muscle tissue and the main pathological hallmarks of laminopathies in LMNA engineered muscle tissue [84,149]. This scaffold-based model has also been used by Chen et al. 2021 [86] to generate a bioengineered 3D skeletal muscle system called “myobundle” to study inflammation in muscle tissue. In this work, the myobundle displayed all the key features of functional skeletal muscle, including electrically or chemically induced twitch and tetanic contractions and robust calcium transients. An IFN-y treatment mimicking chronic inflammation induced myobundle wasting and weakness through the upregulation of the JAK/STAT signaling pathway [86]. By combining the INF-y treatment with exercise-mimicking electrical stimulation or with FDA-approved JAK/STAT inhibitors, the engineered human skeletal muscle showed the ability to counteract inflammation on its own, preventing muscle atrophy by inhibiting the JAK/STAT pathway, suggesting that this tool can also be used for drug screening [86]. Altogether, this model lays the foundation for the study of therapeutic strategies and molecular mechanisms of acquired myopathies, such as idiopathic inflammatory myopathies (IIMs), a heterogeneous group of autoimmune diseases characterized by muscle weakness and inflammation whose pathogenetic mechanisms are still poorly understood [86].

One of the most interesting applications of the organ-on-a-chip technology is the generation of neuromuscular junctions (NMJs). An NMJ, the synaptic connection between a motor nerve and a muscle, is a complex and specialized structure formed by muscle cells, motor neurons (MNs) and Schwann cells [150]. In recent years, human NMJ organoids have been created to achieve the effective innervation of engineered skeletal muscle by co-culturing muscle fibers derived from primary human myoblasts or from iPSCs along with ESC-derived motor neurons using a scaffold-based approach [150,151] or 3D bioprinting technology [7,152]. These models lead to the formation of functional NMJs showing electrical excitability and muscle contractility. An artificial NMJ structure capable of modeling Myasthenia gravis (MG), a rare antibody-mediated autoimmune disease that targets the neuromuscular junction and specifically the postsynaptic muscle end-plate components, was further implemented [85]. In Faustino Martins et al.’s paper, human PSC-derived neuromesodermal progenitors (NMPs) were used to generate a complex, self-organizing neuromuscular organoid containing NMJs supported by terminal Schwann cells and fully functional with muscle contractility, spontaneous calcium oscillations and electrical activities including synchronized neuronal firing. The platform was treated with purified IgG from the sera of MG patients showing a severe impairment of muscle contraction and NMJ integrity, which are the main features of this disease [85]. Bioengineered NMJs generated using a microfluidic device to cultivate 3D myobundles and iPSC-derived MNs from a patient with sporadic Amyotrophic Lateral Sclerosis (ALS) allowed for the disease modeling of an ALS motor unit [151]. The microphysiological 3D ALS motor unit model showed reduced MN viability and muscle contraction strength by optical stimulation and also demonstrated its potential as a drug screening platform, having been tested with ALS drugs administered through an endothelial cell barrier [151]. A complex human sensorimotor organoid containing motor and sensory neurons, astrocytes, microglia, vessels and skeletal muscle fibers was generated from iPSCs obtained from healthy individuals and patients with both familial and sporadic forms of ALS [82]. After 6 weeks of culture, the established sensorimotor organoid formed functional NMJs that resulted in reduced muscle contractions in the ALS-derived organoid model, suggesting a possible NMJ dysfunction in ALS patient-specific iPSCs [82]. A simple but effective and reproducible organoid model for ALS was also established using a scaffold-based technique to form physiologically functional NMJs by co-culturing 3D human skeletal muscle tissues from primary myoblasts together with human iPSC-derived MNs harboring ALS-linked SOD1 mutations. The bioengineered ALS-derived NMJs showed reduced contractions compared to the NMJs from healthy controls, confirming that the 3D neuromuscular cell culture system is suitable for ALS and NMJ disease studies [83].

All these recent studies highlight the great potential of bioengineered tissues to investigate rare diseases with human platforms, maintaining structural and microenvironment complexity combined with the ease of performing high-throughput experiments, genome editing and drug testing. The field is still largely unexplored and needs improvements in terms of reproducibility, but its potential will be further exploited by technological advancements, from organ biofabrication and drug screening platforms to biosensors and monitoring techniques to be integrated in future organ-on-a-chip systems [129,153,154,155].

### 2.3. iPSCs and Organoids for Modeling Bone Disorders 

#### 2.3.1. Bone and Cartilage Organoid Models 

Bone and cartilage, the primary components of the skeletal system, represent specialized forms of connective tissue [156]. Bone is a dynamic organ, and its ability to adapt and change over time is facilitated through the process of bone remodeling. This process is orchestrated by two key types of cells: osteoblasts, which have bone-forming functions and mature into osteocytes, and osteoclasts, responsible for bone resorption [157]. This temporally and spatially synchronized process is intricately regulated by mechanical and chemical stimuli, which trigger various signaling pathways. As a result, replicating this microenvironment in vitro proves to be a challenging task. While 2D cultures are valuable for assessing bone cell functionality, they fall short in elucidating the molecular pathways governing the interplay among bone cells and their interaction with the bone matrix. [158].

Thus, 3D cell aggregates obtained from stem cells have enabled a better understanding of bone mechanisms [159,160]. However, although it has been relatively straightforward to create organoids from cells derived from the brain or liver, the development of bone organoids presents a significant challenge. Bone comprises diverse cell types situated within a unique extracellular matrix (ECM) characterized by a dynamic composition of collagen and minerals. Consequently, organoids generated from mesenchymal stem cells (MSCs) can be regarded as a valuable research model for investigating bone diseases and the identification of potential therapeutic targets [161]. A 3D co-culture of osteoblasts and osteocytes has been generated from the differentiation of stromal cells derived from human bone marrow [162]. This system can be regarded as a three-dimensional in vitro model for studying osteogenesis under both physiological and pathological conditions, as well as for assessing the impact of molecules that affect bone. Nevertheless, mesenchymal stem cells (MSCs) also exhibit certain drawbacks, such as replicative senescence [163], which constrains the potential for obtaining a substantial number of cells.

iPSCs, which can be produced from various adult somatic cell types, allow isogenic disease to be modeled and have been shown to have a high capacity to form cell aggregates [164,165]. Moreover, it is important to note that the generation of bone or cartilage organoids requires not only stem cells but also a matrix scaffold. Nevertheless, using various techniques, certain bone organoid models have been successfully created [166]. For instance, using human iPSCs, an organoid that replicates the bone marrow (referred to as BMO) was developed, comprising hematopoietic, mesenchymal and vascular cells [167]. Furthermore, to investigate interactions between cartilage and bone, O’Connor and colleagues generated an osteochondral organoid using mouse iPSCs by inducing the expression of pluripotency factors through an inducible lentiviral vector [168]. 

#### 2.3.2. Bone and Cartilage Disorders Modeling

The implementation of iPSC-based technology is behind the generation of disease models used to study pathogenetic processes or to identify therapeutic and/or diagnostic targets in the field of skeletal disorders. To elucidate the etiology of juvenile osteochondritis dissecans (JOCDs), a pediatric disease predisposing to early osteoarthritis, Salazar-Noratto conducted a study using iPSCs obtained from JOCDs and control patients [87]. Specifically, iPSCs were obtained from skin biopsies, and mesenchymal stromal cells derived from iPSCs were subsequently differentiated. These cells were analyzed to assess the impact of endoplasmic reticulum stress on chondrogenic and endochondral ossification processes [87]. The researchers further noted that the JOCD cells exhibited a diminished chondrogenic response and impaired endochondral ossification when compared to the control cells. This observation implies an increased susceptibility to stress in JOCD cells. This model can also be considered useful for identifying therapeutic targets in JOCD patients. By using iPSCs obtained from a patient diagnosed with Turner syndrome and with an associated health follow-up, Cui and colleagues observed heightened osteoclastogenesis in the patient’s cells [88]. These findings indicate that the skeletal impairment in Turner syndrome may be attributed to an alteration in the bone resorption process. 

In cartilage derived from iPSCs carrying the heterozygous COL2A1p.G1113C mutation, associated with hypochondrogenesis, the collagen II levels in the extracellular matrix (ECM) were diminished as a result of collagen misfolding at the cellular level [89,90]. The collagen fibrils in the ECM were also disorganized, like those found in human patients and mice with mutations in the Col2a1 gene [169,170]. 

The use of iPSCs could also support studies for the identification of therapeutic targets and/or for drug screening for Osteogenesis Imperfecta (OI), a group of skeletal tissue disorders characterized by bone fragility. When iPSCs carrying the perinatally lethal OI COL1A1p.W1312C mutation were differentiated into chondrocytic and osteoblastic lineages, a notable decrease in collagen I staining within the extracellular matrix (ECM) was observed. This reduction resembled the staining pattern observed in patient-derived fibroblasts [89,91,92]. Kim et al. derived KSCBi006-A iPSCs from peripheral blood mononuclear cells of a patient diagnosed with OI type I through the Sendai virus delivery method. These iPSCs retained the original mutation (COL1A1c.3162delT) and exhibited the capacity to differentiate into all three germ layers [93]. This model, therefore, represents a useful platform for studying OI and for drug screening.

Beyond its utility as a research tool for studying diseases, patient-specific iPSC technology also offers potential for employing restored iPSCs in therapeutic interventions. To this end, Saito et al. generated iPSCs from the primary human oral fibroblasts of patients with cleidocranial dysplasia (CCD), a dominantly inherited skeletal disorder [94]. These CCD iPSCs were generated using retroviral vectors (OCT3/4, SOX2, KLF4 and c-MYC) or a Sendai virus SeVdp vector (KOSM302L), and by applying the CRISPR/Cas9 system, the authors corrected the mutations in the CCD iPSCs [94]. As a result, osteoblasts derived from edited CCD iPSCs and transplanted into rat cranial bone defects exhibited the ability to stimulate bone regeneration, thereby showing the potential therapeutic applications of reprogrammed iPSCs. This last finding suggests that iPSC-based technology is also an innovative tool in regenerative medicine for bone and cartilage disorders.

In conclusion, the ability to reproduce skeletal disease phenotypes in vitro using iPSC-based models promises to improve studies describing the molecular mechanisms of alterations, enabling the identification of therapeutic and diagnostic tools.

### 2.4. Organoids Models for Brain Tumors

Malignant brain tumors are a heterogeneous group of histologically diverse tumors with poor prognosis and high morbidity and mortality rates [171,172]. Patient-derived iPSCs and brain organoids have been employed in cancer research as well as in the field of brain tumors, including glioblastoma, medulloblastoma and meningioma. 

In 2016, Hubert and colleagues succeeded in producing the first glioblastoma 3D organoid culture system, starting directly from patient glioblastoma cells [95]. The authors managed to produce a complex cellular model that housed populations of both stem and non-stem glioblastoma cells, thus recapitulating the complexity present in the donor patient [95]. Instead, Ogawa et al. used CRISPR/Cas9 to model glioma formation in the human ESC cell line H9 by inducing the homologous recombination of an HRasp.G12V-IRES-tdTomato construct into a TP53 locus [51]. Using this approach, it was possible to introduce the activated oncogene HRasp.G12V while simultaneously disrupting the tumor suppressor gene TP53 [51]. The generated organoids displayed tumorigenic and invasive potential in vivo when transplanted into the hippocampus of immunodeficient mice [51]. Recently, a novel approach for generating glioblastoma organoids has arisen. Da Silva et al. developed a protocol in which a patient-derived glioblastoma spheroid was allowed to come into contact with and invade a healthy human cerebral organoid [96]. This model has the advantage of recapitulating the tumor microenvironment in a more accurate way, as glioblastoma cells become embedded in healthy brain tissue [96,173].

A few years later, the first 3D cellular model for group 3 medulloblastoma was produced via the overexpression of the Otx2 and c-MYC genes in human cerebellar organoids [97]. When transplanted into nude mice, the human cerebellar organoids displayed tumorigenic ability and significantly reduced mouse survival [97]. 

Finally, in recent years, two research groups have independently developed protocols for generating meningiomas organoids [99,100]. While Chan and colleagues produced organoids starting from malignant meningioma cell lines (IOMM-Lee and HKBMM), Yamazaki’s approach started directly from patients’ tumors obtained via surgical resection. When comparing the tumor organoid with the patients’ original sample, the histological features and molecular profiles were consistent with those observed in the parental tumors [99,100]. A third patient-derived meningioma organoid model was established to characterize the mechanism underlying the malignancy of high-grade meningiomas. This model fully retains the aggressiveness and the brain invasiveness after an orthotopic transplantation of the unique initiating cell subpopulation and was also used to identify the synthetic compound SRT1720 as a potential agent for systemic treatment and radiation sensitization [158].

Retinoblastoma (Rb) is a rare pediatric cancer of the developing retina in which cancerous cells can sometimes grow along the optic nerve and reach the brain via the optic nerve [174]. Several retinal organoid models have been developed, starting both from human embryonic stem cells and patient-derived iPSCs with germline mutations in the RB transcriptional corepressor 1 (RB1) gene. CRISPR/Cas9 gene editing allowed for the introduction of the desired second RB1 mutation in models obtained from RB1 heterozygous cells or for the full knockout of the RB1 gene. These studies have allowed for an investigation of Rb tumorigenesis, disease modeling, cancer progression and drug testing [101,102,103,104,105]. Retinoblastomas formed from retinal organoids showed high similarity with human cancer and could induce Rb when transplanted into the eyes of immunocompromised mice [103]. When co-cultured with human Rb cells, iPSC-derived retinal organoids resulted in a disorganization of the retinal histoarchitecture in a manner consistent with metastasis and invasion, thus providing a useful ex vivo model system for assessing Rb tumor progression and personalized medicine approaches [175]. These models were also used to test drugs commonly used to treat Rb and screen new ones. For instance, the high-throughput screening of a 133-drug panel resulted in the identification of sutinib, which is FDA-approved for treating renal carcinoma and imatinib–refractory gastrointestinal stromal tumors, as a possible Rb treatment [176].

Overall, in recent years, the development of organoid models to recapitulate brain tumor complexity has received increased attention. Both patient-derived and genetically engineered organoids/iPSCs have displayed phenotypes resembling the clinical behavior of a tumor. Further research is needed to establish these 3D cellular models in other CNS tumors, as well as in other district cancers.

## 3. Conclusions and Future Perspective

Understanding intracellular pathways and alterations in cellular interactions is a challenging obstacle in the study of rare diseases. Not only does the limited number of specimens contribute to the difficulty of the research, but there is also a lack of experimental models that can recapitulate the complex architecture of the tissues involved. In this review, which is not intended to be exhaustive due to the large number of recently developed organoid models, we have chosen to focus on 3D cellular models applied to the study of rare diseases involving neuronal, neuromuscular and skeletal tissues.

The technical approach and potential of the application of 3D cultures derived from human iPSCs in the investigation of cell biology and rare human diseases have been highlighted by the results obtained in the studies commented on in this review. From the analyses of these models, we can categorize the contribution of disease research into the following main areas of interest: (a) alterations in differentiation processes due to the contribution of different cell types (such as MDS and VWR diseases); (b) the characterization of disease subtypes (such as CJD); (c) alterations in the expression of gene clusters involved in cross-cellular pathways (such as HD); (d) potential new therapeutic targets (such as RP, CMT, FTD and IIM diseases); (e) the recapitulation of tissue structural alterations (such as DM) and drug screening (such as OI). 

In the near future, it will be possible to foresee the expansion of applications of cellular models based on human iPSC-derived organoids in genetic studies of rare diseases. Establishing cell models based on a patient’s genetic background may allow for a deeper understanding of disease onset and progression mechanisms. Stem-cell-based organoids may contribute as an efficient in vitro model to analyzing genotype/phenotype correlations at the molecular level, as corroborated by the efficacy of genome editing technologies. The expectations for the future are high, but several limitations of organoids must be mentioned, including their lack of a vascular system, limited differentiation and altered cellular interactions. Standardizing the cell matrix components for 3D cell cultured architectures and carrying out a rigorous validation of cell type differentiation will help achieve consistent and reproducible data to be shared by the scientific community and contribute to novel approaches in gene targeting therapies.

## Figures and Tables

**Table 1:** iPSC/organoid models applied in studying rare neurodegenerative and neuromuscular diseases, brain tumors and skeletal diseases.

Disease	Gene	Cell Model	Reference
NEURODEGENERATIVE/DISORDERS			
Autosomal recessive primary microcephaly (MCPH3) OMIM #604804	CDK5RAP2	iPSC-derived cerebral organoids	[19]
Miller–Dieker Syndrome (MDS; severe form of lissencephaly) OMIM #247200	deletion of 17p13.3 involving LIS1 and YWHAE genes	patient-derived iPSC cortical organoids; patient-specific forebrain organoids + CRISPR/Cas9 genome editing	[45,52]
Leukoencephalopathy with vanishing white matter 1-5 (VWM1-5) OMIM #603896; #620312; #620313; #620314; #620315	EIF2B1-5	iPSC-derived brain organoids with and without eIF2B gene mutations	[66]
Huntington’s disease (HD) OMIM #143100	HTT	iPSC-derived neurons and brain organoids, both carrying HTT mutations	[67]
Sporadic Creutzfeld–Jacob prion disease (CJD) OMIM# 123400	PRNP	cerebral organoids infected with CJD prions	[68]
Retinitis pigmentosa (RP) OMIM#268000	several genes	Patient-derived iPSC retinal organoids; iPSC-derived retinal pigment epithelial cells + CRISPR/Cas9 editing	[69,70,71,72,73,74,75]
Charcot–Marie–Toot 1A (CMT1A) OMIM #118220	PMP22	Patient-derived iPSC PNS organoids	[76]
Axonal Charcot–Marie–Tooth (CMT type 2A1; 2E; 2F; and 2L) OMIM #118210; #607684; #606595; #608373	several genes	iPSC-derived neurons from CMT2 patients + CRISPR/Cas9 editing	[77]
Frontotemporal dementia (FTD) with or without Parkinsonism OMIM#600274	MAPT	iPSC-derived cerebral organoids from patients carrying MAPT mutations; patient-derived iPSC lines with MAPT mutations + CRISPR/Cas9 editing	[78,79,80]
NEUROMUSCULAR DISORDERS			
Amyotrophic lateral sclerosis overlapping with frontotemporal dementia (ALS/FTD) OMIM# 612069	TARDBP (TDP43)	patient-derived iPSC cortical organoids iPSC-derived co-cultured neurons and astrocytes	[81]
Amyotrophic lateral sclerosis (ALS) OMIM #105400	SOD1	patient-derived iPSC sensorimotor organoids; ALS iPSCs with SOD1 mutations and primary myoblasts co-culture	[82,83]
Duchenne muscular dystrophy (DMD) OMIM #310200	DMD	patient-derived iPSC skeletal muscle organoids + CRISPR/Cas9 editing; 3D co-culture	[84]
Limb–girdle muscular dystrophy type R3 (LGMDR3) OMIM# 608099	SGCA	3D co-culture	[84]
LAMIN A/C (LMNA)-related muscular dystrophies OMIM# 613205	LMNA	3D co-culture	[84]
Myasthenia gravis (MG) OMIM #254200	---	human iPSC-derived neuromuscular organoids containing neuromuscular junctions	[85]
Idiopathic inflammatory myopathies	---	3D myobundle	[86]
SKELETAL DISORDERS			
Juvenile osteochondritis disseccans (JOCD)	---	patient-derived iPSC lines, 3D chondrogenic modeling	[87]
Turner syndrome	X chromosome	patient-derived iPSC lines	[88]
Hypochondrogenesis (achondrogenesis type II) OMIM #200610	COL2A1	cartilage produced from patient-derived iPSCs	[89,90]
Osteogenesis imperfecta (OI) OMIM#120150	COL1A1	patient-derived iPSC lines	[91,92,93]
Cleidocranial dysplasia (CCD) OMIM # 119600	RUNX2	patient-derived iPSC lines + CRISPR/Cas9 editing	[94]
BRAIN TUMORS			
Glioblastoma (GB)	HRAS TP53	patient-derived glioblastoma organoids; human-ESC-derived glioma + CRISPR/Cas9 editing; glioblastoma spheroid invasion of a brain organoid (GLICO)	[51,95,96]
Medulloblastoma (MB)	Otx2/c-MYC	genetically engineered iPSC cerebellar organoids	[97]
Meningioma	---	patient-derived meningioma organoids	[98,99,100]
Retinoblastoma	RB1	human-ESC-derived and patient-derived retinal organoids + CRISPR/Cas9 editing	[101,102,103,104,105]

## References

[1] Novelli G., Spitalieri P., Murdocca M., Centanini E., Sangiuolo F. (2022). Organoid Factory: The Recent Role of the Human Induced Pluripotent Stem Cells (hiPSCs) in Precision Medicine. Front. Cell Dev. Biol..

[2] Ma M., Moulton M.J., Lu S., Bellen H.J. (2022). “Fly-Ing” from Rare to Common Neurodegenerative Disease Mechanisms. Trends Genet. TIG.

[3] Adamson K.I., Sheridan E., Grierson A.J. (2018). Use of Zebrafish Models to Investigate Rare Human Disease. J. Med. Genet..

[4] Perlman R.L. (2016). Mouse Models of Human Disease: An Evolutionary Perspective. Evol. Med. Public Health.

[5] Bai X. (2020). Stem Cell-Based Disease Modeling and Cell Therapy. Cells.

[6] Argentati C., Tortorella I., Bazzucchi M., Morena F., Martino S. (2020). Harnessing the Potential of Stem Cells for Disease Modeling: Progress and Promises. J. Pers. Med..

[7] Kim J., Koo B.-K., Knoblich J.A. (2020). Human Organoids: Model Systems for Human Biology and Medicine. Nat. Rev. Mol. Cell Biol..

[8] Corrò C., Novellasdemunt L., Li V.S.W. (2020). A Brief History of Organoids. Am. J. Physiol. Cell Physiol..

[9] Silva-Pedrosa R., Salgado A.J., Ferreira P.E. (2023). Revolutionizing Disease Modeling: The Emergence of Organoids in Cellular Systems. Cells.

[10] Andrews M.G., Kriegstein A.R. (2022). Challenges of Organoid Research. Annu. Rev. Neurosci..

[11] Calà G., Sina B., De Coppi P., Giobbe G.G., Gerli M.F.M. (2023). Primary Human Organoids Models: Current Progress and Key Milestones. Front. Bioeng. Biotechnol..

[12] Zhao Z., Chen X., Dowbaj A.M., Sljukic A., Bratlie K., Lin L., Fong E.L.S., Balachander G.M., Chen Z., Soragni A. (2022). Organoids. Nat. Rev. Methods Primers.

[13] Teriyapirom I., Batista-Rocha A.S., Koo B.-K. (2021). Genetic Engineering in Organoids. J. Mol. Med. Berl. Ger..

[14] Huch M., Koo B.-K. (2015). Modeling Mouse and Human Development Using Organoid Cultures. Dev. Camb. Engl..

[15] Lancaster M.A., Knoblich J.A. (2014). Generation of Cerebral Organoids from Human Pluripotent Stem Cells. Nat. Protoc..

[16] Chao Y., Xiang Y., Xiao J., Zheng W., Ebrahimkhani M.R., Yang C., Wu A.R., Liu P., Huang Y., Sugimura R. (2023). Organoid-Based Single-Cell Spatiotemporal Gene Expression Landscape of Human Embryonic Development and Hematopoiesis. Signal Transduct. Target. Ther..

[17] Wagstaff E.L., Heredero Berzal A., Boon C.J.F., Quinn P.M.J., Ten Asbroek A.L.M.A., Bergen A.A. (2021). The Role of Small Molecules and Their Effect on the Molecular Mechanisms of Early Retinal Organoid Development. Int. J. Mol. Sci..

[18] Schutgens F., Clevers H. (2020). Human Organoids: Tools for Understanding Biology and Treating Diseases. Annu. Rev. Pathol..

[19] Lancaster M.A., Renner M., Martin C.-A., Wenzel D., Bicknell L.S., Hurles M.E., Homfray T., Penninger J.M., Jackson A.P., Knoblich J.A. (2013). Cerebral Organoids Model Human Brain Development and Microcephaly. Nature.

[20] McCracken K.W., Catá E.M., Crawford C.M., Sinagoga K.L., Schumacher M., Rockich B.E., Tsai Y.-H., Mayhew C.N., Spence J.R., Zavros Y. (2014). Modelling Human Development and Disease in Pluripotent Stem-Cell-Derived Gastric Organoids. Nature.

[21] Spence J.R., Mayhew C.N., Rankin S.A., Kuhar M.F., Vallance J.E., Tolle K., Hoskins E.E., Kalinichenko V.V., Wells S.I., Zorn A.M. (2011). Directed Differentiation of Human Pluripotent Stem Cells into Intestinal Tissue In Vitro. Nature.

[22] Rothenbücher T.S.P., Gürbüz H., Pereira M.P., Heiskanen A., Emneus J., Martinez-Serrano A. (2021). Next Generation Human Brain Models: Engineered Flat Brain Organoids Featuring Gyrification. Biofabrication.

[23] Rispoli P., Scandiuzzi Piovesan T., Decorti G., Stocco G., Lucafò M. (2023). iPSCs as a Groundbreaking Tool for the Study of Adverse Drug Reactions: A New Avenue for Personalized Therapy. WIREs Mech. Dis..

[24] Liu Y., Chen Y.-G. (2018). 2D- and 3D-Based Intestinal Stem Cell Cultures for Personalized Medicine. Cells.

[25] Pérez-González C., Ceada G., Greco F., Matejčić M., Gómez-González M., Castro N., Menendez A., Kale S., Krndija D., Clark A.G. (2021). Mechanical Compartmentalization of the Intestinal Organoid Enables Crypt Folding and Collective Cell Migration. Nat. Cell Biol..

[26] Kilpatrick S., Irwin C., Singh K.K. (2023). Human Pluripotent Stem Cell (hPSC) and Organoid Models of Autism: Opportunities and Limitations. Transl. Psychiatry.

[27] Song G., Zhao M., Chen H., Zhou X., Lenahan C., Ou Y., He Y. (2021). The Application of Brain Organoid Technology in Stroke Research: Challenges and Prospects. Front. Cell. Neurosci..

[28] Wang Z., Wang S.-N., Xu T.-Y., Miao Z.-W., Su D.-F., Miao C.-Y. (2017). Organoid Technology for Brain and Therapeutics Research. CNS Neurosci. Ther..

[29] Yang H. (2022). Tau and Stathmin Proteins in Breast Cancer: A Potential Therapeutic Target. Clin. Exp. Pharmacol. Physiol..

[30] Xiang K., Zhuang H. (2023). Liver Organoid Potential Application for Hepatitis E Virus Infection. Adv. Exp. Med. Biol..

[31] Heo I., Dutta D., Schaefer D.A., Iakobachvili N., Artegiani B., Sachs N., Boonekamp K.E., Bowden G., Hendrickx A.P.A., Willems R.J.L. (2018). Modelling Cryptosporidium Infection in Human Small Intestinal and Lung Organoids. Nat. Microbiol..

[32] Artegiani B., Clevers H. (2018). Use and Application of 3D-Organoid Technology. Hum. Mol. Genet..

[33] Xu M., Lee E.M., Wen Z., Cheng Y., Huang W.-K., Qian X., Tcw J., Kouznetsova J., Ogden S.C., Hammack C. (2016). Identification of Small-Molecule Inhibitors of Zika Virus Infection and Induced Neural Cell Death via a Drug Repurposing Screen. Nat. Med..

[34] Perrone F., Zilbauer M. (2021). Biobanking of Human Gut Organoids for Translational Research. Exp. Mol. Med..

[35] Broutier L., Andersson-Rolf A., Hindley C.J., Boj S.F., Clevers H., Koo B.-K., Huch M. (2016). Culture and Establishment of Self-Renewing Human and Mouse Adult Liver and Pancreas 3D Organoids and Their Genetic Manipulation. Nat. Protoc..

[36] Munro M.J., Tan S.T., Gray C. (2023). Applications for Colon Organoid Models in Cancer Research. Organoids.

[37] Flood P., Hanrahan N., Nally K., Melgar S. (2023). Human Intestinal Organoids: Modeling Gastrointestinal Physiology and Immunopathology—Current Applications and Limitations. Eur. J. Immunol..

[38] Di Giorgio C., Roselli R., Biagioli M., Bordoni M., Ricci P., Zampella A., Distrutti E., Donini A., Fiorucci S. (2023). Modeling Inflammatory Bowel Disease by Intestinal Organoids. Recent Adv. Inflamm. Allergy Drug Discov..

[39] Yang C., Xiao W., Wang R., Hu Y., Yi K., Sun X., Wang G., Xu X. (2023). Tumor Organoid Model of Colorectal Cancer (Review). Oncol. Lett..

[40] Berkers G., van Mourik P., Vonk A.M., Kruisselbrink E., Dekkers J.F., de Winter-de Groot K.M., Arets H.G.M., Marck-van der Wilt R.E.P., Dijkema J.S., Vanderschuren M.M. (2019). Rectal Organoids Enable Personalized Treatment of Cystic Fibrosis. Cell Rep..

[41] Del Dosso A., Urenda J.-P., Nguyen T., Quadrato G. (2020). Upgrading the Physiological Relevance of Human Brain Organoids. Neuron.

[42] Wang L., Ye Z., Jang Y.-Y. (2021). Convergence of Human Pluripotent Stem Cell, Organoid, and Genome Editing Technologies. Exp. Biol. Med..

[43] Fischer J., Heide M., Huttner W.B. (2019). Genetic Modification of Brain Organoids. Front. Cell. Neurosci..

[44] Deverman B.E., Pravdo P.L., Simpson B.P., Kumar S.R., Chan K.Y., Banerjee A., Wu W.-L., Yang B., Huber N., Pasca S.P. (2016). Cre-Dependent Selection Yields AAV Variants for Widespread Gene Transfer to the Adult Brain. Nat. Biotechnol..

[45] Bershteyn M., Nowakowski T.J., Pollen A.A., Di Lullo E., Nene A., Wynshaw-Boris A., Kriegstein A.R. (2017). Human iPSC-Derived Cerebral Organoids Model Cellular Features of Lissencephaly and Reveal Prolonged Mitosis of Outer Radial Glia. Cell Stem Cell.

[46] Birey F., Andersen J., Makinson C.D., Islam S., Wei W., Huber N., Fan H.C., Metzler K.R.C., Panagiotakos G., Thom N. (2017). Assembly of Functionally Integrated Human Forebrain Spheroids. Nature.

[47] Schwank G., Koo B.-K., Sasselli V., Dekkers J.F., Heo I., Demircan T., Sasaki N., Boymans S., Cuppen E., van der Ent C.K. (2013). Functional Repair of CFTR by CRISPR/Cas9 in Intestinal Stem Cell Organoids of Cystic Fibrosis Patients. Cell Stem Cell.

[48] Kawasaki K., Fujii M., Sugimoto S., Ishikawa K., Matano M., Ohta Y., Toshimitsu K., Takahashi S., Hosoe N., Sekine S. (2020). Chromosome Engineering of Human Colon-Derived Organoids to Develop a Model of Traditional Serrated Adenoma. Gastroenterology.

[49] Matano M., Date S., Shimokawa M., Takano A., Fujii M., Ohta Y., Watanabe T., Kanai T., Sato T. (2015). Modeling Colorectal Cancer Using CRISPR-Cas9-Mediated Engineering of Human Intestinal Organoids. Nat. Med..

[50] Dekkers J.F., Whittle J.R., Vaillant F., Chen H.-R., Dawson C., Liu K., Geurts M.H., Herold M.J., Clevers H., Lindeman G.J. (2020). Modeling Breast Cancer Using CRISPR-Cas9-Mediated Engineering of Human Breast Organoids. J. Natl. Cancer Inst..

[51] Ogawa J., Pao G.M., Shokhirev M.N., Verma I.M. (2018). Glioblastoma Model Using Human Cerebral Organoids. Cell Rep..

[52] Iefremova V., Manikakis G., Krefft O., Jabali A., Weynans K., Wilkens R., Marsoner F., Brändl B., Müller F.-J., Koch P. (2017). An Organoid-Based Model of Cortical Development Identifies Non-Cell-Autonomous Defects in Wnt Signaling Contributing to Miller-Dieker Syndrome. Cell Rep..

[53] Matsui T., Nieto-Estévez V., Kyrychenko S., Schneider J.W., Hsieh J. (2017). Retinoblastoma Protein Controls Growth, Survival and Neuronal Migration in Human Cerebral Organoids. Dev. Camb. Engl..

[54] Fiddes I.T., Lodewijk G.A., Mooring M., Bosworth C.M., Ewing A.D., Mantalas G.L., Novak A.M., van den Bout A., Bishara A., Rosenkrantz J.L. (2018). Human-Specific NOTCH2NL Genes Affect Notch Signaling and Cortical Neurogenesis. Cell.

[55] Karzbrun E., Kshirsagar A., Cohen S.R., Hanna J.H., Reiner O. (2018). Human Brain Organoids on a Chip Reveal the Physics of Folding. Nat. Phys..

[56] Xiaoshuai L., Qiushi W., Rui W. (2022). Advantages of CRISPR-Cas9 Combined Organoid Model in the Study of Congenital Nervous System Malformations. Front. Bioeng. Biotechnol..

[57] Inak G., Rybak-Wolf A., Lisowski P., Pentimalli T.M., Jüttner R., Glažar P., Uppal K., Bottani E., Brunetti D., Secker C. (2021). Defective Metabolic Programming Impairs Early Neuronal Morphogenesis in Neural Cultures and an Organoid Model of Leigh Syndrome. Nat. Commun..

[58] Bendriem R.M., Singh S., Aleem A.A., Antonetti D.A., Ross M.E. (2019). Tight Junction Protein Occludin Regulates Progenitor Self-Renewal and Survival in Developing Cortex. eLife.

[59] Bhatia S., Pooja, Yadav S.K. (2023). CRISPR-Cas for Genome Editing: Classification, Mechanism, Designing and Applications. Int. J. Biol. Macromol..

[60] Liu G., Zhang Y., Zhang T. (2020). Computational Approaches for Effective CRISPR Guide RNA Design and Evaluation. Comput. Struct. Biotechnol. J..

[61] Kang S.-Y., Kimura M., Shrestha S., Lewis P., Lee S., Cai Y., Joshi P., Acharya P., Liu J., Yang Y. (2023). A Pillar and Perfusion Plate Platform for Robust Human Organoid Culture and Analysis. Adv. Healthc. Mater..

[62] Richardson D.S., Lichtman J.W. (2015). Clarifying Tissue Clearing. Cell.

[63] Zhang Y.S., Aleman J., Shin S.R., Kilic T., Kim D., Mousavi Shaegh S.A., Massa S., Riahi R., Chae S., Hu N. (2017). Multisensor-Integrated Organs-on-Chips Platform for Automated and Continual in Situ Monitoring of Organoid Behaviors. Proc. Natl. Acad. Sci. USA.

[64] Takebe T., Sekine K., Kimura M., Yoshizawa E., Ayano S., Koido M., Funayama S., Nakanishi N., Hisai T., Kobayashi T. (2017). Massive and Reproducible Production of Liver Buds Entirely from Human Pluripotent Stem Cells. Cell Rep..

[65] Takebe T., Zhang R.-R., Koike H., Kimura M., Yoshizawa E., Enomura M., Koike N., Sekine K., Taniguchi H. (2014). Generation of a Vascularized and Functional Human Liver from an iPSC-Derived Organ Bud Transplant. Nat. Protoc..

[66] Deng J., Zhang J., Gao K., Zhou L., Jiang Y., Wang J., Wu Y. (2023). Human-Induced Pluripotent Stem Cell-Derived Cerebral Organoid of Leukoencephalopathy with Vanishing White Matter. CNS Neurosci. Ther..

[67] Faravelli I., Costamagna G., Tamanini S., Corti S. (2020). Back to the Origins: Human Brain Organoids to Investigate Neurodegeneration. Brain Res..

[68] Groveman B.R., Foliaki S.T., Orru C.D., Zanusso G., Carroll J.A., Race B., Haigh C.L. (2019). Sporadic Creutzfeldt-Jakob Disease Prion Infection of Human Cerebral Organoids. Acta Neuropathol. Commun..

[69] Gao M.-L., Lei X.-L., Han F., He K.-W., Jin S.-Q., Zhang Y.-Y., Jin Z.-B. (2020). Patient-Specific Retinal Organoids Recapitulate Disease Features of Late-Onset Retinitis Pigmentosa. Front. Cell Dev. Biol..

[70] Su T., Liang L., Zhang L., Wang J., Chen L., Su C., Cao J., Yu Q., Deng S., Chan H.F. (2022). Retinal Organoids and Microfluidic Chip-Based Approaches to Explore the Retinitis Pigmentosa with USH2A Mutations. Front. Bioeng. Biotechnol..

[71] Zhang X., Wang W., Jin Z.-B. (2021). Retinal Organoids as Models for Development and Diseases. Cell Regen..

[72] Deng W.-L., Gao M.-L., Lei X.-L., Lv J.-N., Zhao H., He K.-W., Xia X.-X., Li L.-Y., Chen Y.-C., Li Y.-P. (2018). Gene Correction Reverses Ciliopathy and Photoreceptor Loss in iPSC-Derived Retinal Organoids from Retinitis Pigmentosa Patients. Stem Cell Rep..

[73] Li Y.-P., Deng W.-L., Jin Z.-B. (2021). Modeling Retinitis Pigmentosa through Patient-Derived Retinal Organoids. STAR Protoc..

[74] Kandoi S., Martinez C., Chen K.X., Mehine M., Mansfield B.C., Duncan J.L., Lamba D.A. (2023). Disease Modeling and Pharmacological Rescue of Autosomal Dominant Retinitis Pigmentosa Associated with RHO Copy Number Variation. medRxiv.

[75] Rodrigues A., Slembrouck-Brec A., Nanteau C., Terray A., Tymoshenko Y., Zagar Y., Reichman S., Xi Z., Sahel J.-A., Fouquet S. (2022). Modeling PRPF31 Retinitis Pigmentosa Using Retinal Pigment Epithelium and Organoids Combined with Gene Augmentation Rescue. NPJ Regen. Med..

[76] Van Lent J., Vendredy L., Adriaenssens E., Da Silva Authier T., Asselbergh B., Kaji M., Weckhuysen S., Van Den Bosch L., Baets J., Timmerman V. (2023). Downregulation of PMP22 Ameliorates Myelin Defects in iPSC-Derived Human Organoid Cultures of CMT1A. Brain.

[77] Van Lent J., Verstraelen P., Asselbergh B., Adriaenssens E., Mateiu L., Verbist C., De Winter V., Eggermont K., Van Den Bosch L., De Vos W.H. (2021). Induced Pluripotent Stem Cell-Derived Motor Neurons of CMT Type 2 Patients Reveal Progressive Mitochondrial Dysfunction. Brain.

[78] Bowles K.R., Silva M.C., Whitney K., Bertucci T., Berlind J.E., Lai J.D., Garza J.C., Boles N.C., Mahali S., Strang K.H. (2021). ELAVL4, Splicing, and Glutamatergic Dysfunction Precede Neuron Loss in MAPT Mutation Cerebral Organoids. Cell.

[79] Glasauer S.M.K., Goderie S.K., Rauch J.N., Guzman E., Audouard M., Bertucci T., Joy S., Rommelfanger E., Luna G., Keane-Rivera E. (2022). Human Tau Mutations in Cerebral Organoids Induce a Progressive Dyshomeostasis of Cholesterol. Stem Cell Rep..

[80] Lines G., Casey J.M., Preza E., Wray S. (2020). Modelling Frontotemporal Dementia Using Patient-Derived Induced Pluripotent Stem Cells. Mol. Cell. Neurosci..

[81] de Majo M., Koontz M., Marsan E., Salinas N., Ramsey A., Kuo Y.-M., Seo K., Li H., Dräger N., Leng K. (2023). Granulin Loss of Function in Human Mature Brain Organoids Implicates Astrocytes in TDP-43 Pathology. Stem Cell Rep..

[82] Pereira J.D., DuBreuil D.M., Devlin A.-C., Held A., Sapir Y., Berezovski E., Hawrot J., Dorfman K., Chander V., Wainger B.J. (2021). Human Sensorimotor Organoids Derived from Healthy and Amyotrophic Lateral Sclerosis Stem Cells Form Neuromuscular Junctions. Nat. Commun..

[83] Massih B., Veh A., Schenke M., Mungwa S., Seeger B., Selvaraj B.T., Chandran S., Reinhardt P., Sterneckert J., Hermann A. (2023). A 3D Cell Culture System for Bioengineering Human Neuromuscular Junctions to Model ALS. Front. Cell Dev. Biol..

[84] Maffioletti S.M., Sarcar S., Henderson A.B.H., Mannhardt I., Pinton L., Moyle L.A., Steele-Stallard H., Cappellari O., Wells K.E., Ferrari G. (2018). Three-Dimensional Human iPSC-Derived Artificial Skeletal Muscles Model Muscular Dystrophies and Enable Multilineage Tissue Engineering. Cell Rep..

[85] Faustino Martins J.-M., Fischer C., Urzi A., Vidal R., Kunz S., Ruffault P.-L., Kabuss L., Hube I., Gazzerro E., Birchmeier C. (2020). Self-Organizing 3D Human Trunk Neuromuscular Organoids. Cell Stem Cell.

[86] Chen Z., Li B., Zhan R.-Z., Rao L., Bursac N. (2021). Exercise Mimetics and JAK Inhibition Attenuate IFN-γ-Induced Wasting in Engineered Human Skeletal Muscle. Sci. Adv..

[87] Salazar-Noratto G.E., Nations C.C., Stevens H.Y., Xu M., Gaynard S., Dooley C., de Nijs N., McDonagh K., Shen S., Willimon S.C. (2023). Patient-Specific iPSC-Derived Models Link Aberrant Endoplasmic Reticulum Stress Sensing and Response to Juvenile Osteochondritis Dissecans Etiology. Stem Cells Transl. Med..

[88] Cui X., Cui Y., Shi L., Luan J., Zhou X., Han J. (2019). A Preliminary Study on the Mechanism of Skeletal Abnormalities in Turner Syndrome Using Inducing Pluripotent Stem Cells (iPS)-Based Disease Models. Intractable Rare Dis. Res..

[89] Lamandé S.R., Ng E.S., Cameron T.L., Kung L.H.W., Sampurno L., Rowley L., Lilianty J., Patria Y.N., Stenta T., Hanssen E. (2023). Modeling Human Skeletal Development Using Human Pluripotent Stem Cells. Proc. Natl. Acad. Sci. USA.

[90] Lilianty J., Bateman J.F., Lamandé S.R. (2021). Generation of a Heterozygous COL2A1 (p.G1113C) Hypochondrogenesis Mutation iPSC Line, MCRIi019-A-7, Using CRISPR/Cas9 Gene Editing. Stem Cell Res..

[91] Howden S., Hosseini Far H., Motazedian A., Elefanty A.G., Stanley E.G., Lamandé S.R., Bateman J.F. (2019). The Use of Simultaneous Reprogramming and Gene Correction to Generate an Osteogenesis Imperfecta Patient COL1A1 c. 3936 G>T iPSC Line and an Isogenic Control iPSC Line. Stem Cell Res..

[92] Lamandé S.R., Chessler S.D., Golub S.B., Byers P.H., Chan D., Cole W.G., Sillence D.O., Bateman J.F. (1995). Endoplasmic Reticulum-Mediated Quality Control of Type I Collagen Production by Cells from Osteogenesis Imperfecta Patients with Mutations in the pro Alpha 1 (I) Chain Carboxyl-Terminal Propeptide Which Impair Subunit Assembly. J. Biol. Chem..

[93] Kim B.-Y., Ko J.M., Park M.-H., Koo S.K. (2019). Generation of a Patient-Specific Induced Pluripotent Stem Cell Line, KSCBi006-A, for Osteogenesis Imperfecta Type I with the COL1A1, c.3162delT Mutation. Stem Cell Res..

[94] Saito A., Ooki A., Nakamura T., Onodera S., Hayashi K., Hasegawa D., Okudaira T., Watanabe K., Kato H., Onda T. (2018). Targeted Reversion of Induced Pluripotent Stem Cells from Patients with Human Cleidocranial Dysplasia Improves Bone Regeneration in a Rat Calvarial Bone Defect Model. Stem Cell Res. Ther..

[95] Hubert C.G., Rivera M., Spangler L.C., Wu Q., Mack S.C., Prager B.C., Couce M., McLendon R.E., Sloan A.E., Rich J.N. (2016). A Three-Dimensional Organoid Culture System Derived from Human Glioblastomas Recapitulates the Hypoxic Gradients and Cancer Stem Cell Heterogeneity of Tumors Found In Vivo. Cancer Res..

[96] da Silva B., Mathew R.K., Polson E.S., Williams J., Wurdak H. (2018). Spontaneous Glioblastoma Spheroid Infiltration of Early-Stage Cerebral Organoids Models Brain Tumor Invasion. SLAS Discov. Adv. Life Sci. R D.

[97] Ballabio C., Anderle M., Gianesello M., Lago C., Miele E., Cardano M., Aiello G., Piazza S., Caron D., Gianno F. (2020). Modeling Medulloblastoma In Vivo and with Human Cerebellar Organoids. Nat. Commun..

[98] Huang M., Xu S., Li Y., Shang L., Zhan X., Qin C., Su J., Zhao Z., He Y., Qin L. (2023). Novel Human Meningioma Organoids Recapitulate the Aggressiveness of the Initiating Cell Subpopulations Identified by ScRNA-Seq. Adv. Sci..

[99] Chan H.S.C., Ng H.K., Chan A.K.-Y., Cheng S.H., Chow C., Wong N., Wong G.K.C. (2021). Establishment and Characterization of Meningioma Patient-Derived Organoid. J. Clin. Neurosci. Off. J. Neurosurg. Soc. Australas..

[100] Yamazaki S., Ohka F., Hirano M., Shiraki Y., Motomura K., Tanahashi K., Tsujiuchi T., Motomura A., Aoki K., Shinjo K. (2021). Newly Established Patient-Derived Organoid Model of Intracranial Meningioma. Neuro-Oncol..

[101] Li Y.-P., Wang Y.-T., Wang W., Zhang X., Shen R.-J., Jin K., Jin L.-W., Jin Z.-B. (2022). Second Hit Impels Oncogenesis of Retinoblastoma in Patient-Induced Pluripotent Stem Cell-Derived Retinal Organoids: Direct Evidence for Knudson’s Theory. PNAS Nexus.

[102] Liu H., Zhang Y., Zhang Y.-Y., Li Y.-P., Hua Z.-Q., Zhang C.-J., Wu K.-C., Yu F., Zhang Y., Su J. (2020). Human Embryonic Stem Cell-Derived Organoid Retinoblastoma Reveals a Cancerous Origin. Proc. Natl. Acad. Sci. USA.

[103] Norrie J.L., Nityanandam A., Lai K., Chen X., Wilson M., Stewart E., Griffiths L., Jin H., Wu G., Orr B. (2021). Retinoblastoma from Human Stem Cell-Derived Retinal Organoids. Nat. Commun..

[104] Cheng Y.-M., Ma C., Jin K., Jin Z.-B. (2023). Retinal Organoid and Gene Editing for Basic and Translational Research. Vision Res..

[105] Rozanska A., Cerna-Chavez R., Queen R., Collin J., Zerti D., Dorgau B., Beh C.S., Davey T., Coxhead J., Hussain R. (2022). pRB-Depleted Pluripotent Stem Cell Retinal Organoids Recapitulate Cell State Transitions of Retinoblastoma Development and Suggest an Important Role for pRB in Retinal Cell Differentiation. Stem Cells Transl. Med..

[106] Mulder L.A., Depla J.A., Sridhar A., Wolthers K., Pajkrt D., Vieira de Sá R. (2023). A Beginner’s Guide on the Use of Brain Organoids for Neuroscientists: A Systematic Review. Stem Cell Res. Ther..

[107] Wynshaw-Boris A. (2007). Lissencephaly and LIS1: Insights into the Molecular Mechanisms of Neuronal Migration and Development. Clin. Genet..

[108] Hamilton E.M.C., van der Lei H.D.W., Vermeulen G., Gerver J.A.M., Lourenço C.M., Naidu S., Mierzewska H., Gemke R.J.B.J., de Vet H.C.W., Uitdehaag B.M.J. (2018). Natural History of Vanishing White Matter. Ann. Neurol..

[109] Trimouille A., Marguet F., Sauvestre F., Lasseaux E., Pelluard F., Martin-Négrier M.-L., Plaisant C., Rooryck C., Lacombe D., Arveiler B. (2020). Foetal Onset of EIF2B Related Disorder in Two Siblings: Cerebellar Hypoplasia with Absent Bergmann Glia and Severe Hypomyelination. Acta Neuropathol. Commun..

[110] Conforti P., Besusso D., Bocchi V.D., Faedo A., Cesana E., Rossetti G., Ranzani V., Svendsen C.N., Thompson L.M., Toselli M. (2018). Faulty Neuronal Determination and Cell Polarization Are Reverted by Modulating HD Early Phenotypes. Proc. Natl. Acad. Sci. USA.

[111] The HD iPSC Consortium (2017). Developmental Alterations in Huntington’s Disease Neural Cells and Pharmacological Rescue in Cells and Mice. Nat. Neurosci..

[112] Mehta S.R., Tom C.M., Wang Y., Bresee C., Rushton D., Mathkar P.P., Tang J., Mattis V.B. (2018). Human Huntington’s Disease iPSC-Derived Cortical Neurons Display Altered Transcriptomics, Morphology, and Maturation. Cell Rep..

[113] Groveman B.R., Race B., Foliaki S.T., Williams K., Hughson A.G., Baune C., Zanusso G., Haigh C.L. (2023). Sporadic Creutzfeldt–Jakob Disease Infected Human Cerebral Organoids Retain the Original Human Brain Subtype Features Following Transmission to Humanized Transgenic Mice. Acta Neuropathol. Commun..

[114] Okamoto Y., Takashima H. (2023). The Current State of Charcot–Marie–Tooth Disease Treatment. Genes.

[115] Mansour A.A., Gonçalves J.T., Bloyd C.W., Li H., Fernandes S., Quang D., Johnston S., Parylak S.L., Jin X., Gage F.H. (2018). An in Vivo Model of Functional and Vascularized Human Brain Organoids. Nat. Biotechnol..

[116] Uzquiano A., Kedaigle A.J., Pigoni M., Paulsen B., Adiconis X., Kim K., Faits T., Nagaraja S., Antón-Bolaños N., Gerhardinger C. (2022). Proper Acquisition of Cell Class Identity in Organoids Allows Definition of Fate Specification Programs of the Human Cerebral Cortex. Cell.

[117] Velasco S. (2022). Modeling Brain Disorders Using Transplanted Organoids: Beyond the Short Circuit. Cell Stem Cell.

[118] Yoon S.-J., Elahi L.S., Pașca A.M., Marton R.M., Gordon A., Revah O., Miura Y., Walczak E.M., Holdgate G.M., Fan H.C. (2019). Reliability of Human Cortical Organoid Generation. Nat. Methods.

[119] Szebényi K., Wenger L.M.D., Sun Y., Dunn A.W.E., Limegrover C.A., Gibbons G.M., Conci E., Paulsen O., Mierau S.B., Balmus G. (2021). Human ALS/FTD Brain Organoid Slice Cultures Display Distinct Early Astrocyte and Targetable Neuronal Pathology. Nat. Neurosci..

[120] Corsi A., Bombieri C., Valenti M.T., Romanelli M.G. (2022). Tau Isoforms: Gaining Insight into MAPT Alternative Splicing. Int. J. Mol. Sci..

[121] Bertucci T., Bowles K.R., Lotz S., Qi L., Stevens K., Goderie S.K., Borden S., Oja L.M., Lane K., Lotz R. (2023). Improved Protocol for Reproducible Human Cortical Organoids Reveals Early Alterations in Metabolism with MAPT Mutations. BioRxiv.

[122] Nakamura M., Shiozawa S., Tsuboi D., Amano M., Watanabe H., Maeda S., Kimura T., Yoshimatsu S., Kisa F., Karch C.M. (2019). Pathological Progression Induced by the Frontotemporal Dementia-Associated R406W Tau Mutation in Patient-Derived iPSCs. Stem Cell Rep..

[123] Seo J., Kritskiy O., Watson L.A., Barker S.J., Dey D., Raja W.K., Lin Y.-T., Ko T., Cho S., Penney J. (2017). Inhibition of P25/Cdk5 Attenuates Tauopathy in Mouse and iPSC Models of Frontotemporal Dementia. J. Neurosci. Off. J. Soc. Neurosci..

[124] Iberite F., Gruppioni E., Ricotti L. (2022). Skeletal Muscle Differentiation of Human iPSCs Meets Bioengineering Strategies: Perspectives and Challenges. NPJ Regen. Med..

[125] Jalal S., Dastidar S., Tedesco F.S. (2021). Advanced Models of Human Skeletal Muscle Differentiation, Development and Disease: Three-Dimensional Cultures, Organoids and Beyond. Curr. Opin. Cell Biol..

[126] Ray A., Joshi J.M., Sundaravadivelu P.K., Raina K., Lenka N., Kaveeshwar V., Thummer R.P. (2021). An Overview on Promising Somatic Cell Sources Utilized for the Efficient Generation of Induced Pluripotent Stem Cells. Stem Cell Rev. Rep..

[127] Maffioletti S.M., Gerli M.F.M., Ragazzi M., Dastidar S., Benedetti S., Loperfido M., VandenDriessche T., Chuah M.K., Tedesco F.S. (2015). Efficient Derivation and Inducible Differentiation of Expandable Skeletal Myogenic Cells from Human ES and Patient-Specific iPS Cells. Nat. Protoc..

[128] Rao L., Qian Y., Khodabukus A., Ribar T., Bursac N. (2018). Engineering Human Pluripotent Stem Cells into a Functional Skeletal Muscle Tissue. Nat. Commun..

[129] Fernández-Costa J.M., Tejedera-Vilafranca A., Fernández-Garibay X., Ramón-Azcón J. (2023). Muscle-on-a-Chip Devices: A New Era for in Vitro Modelling of Muscular Dystrophies. Dis. Model. Mech..

[130] Pinton L., Khedr M., Lionello V.M., Sarcar S., Maffioletti S.M., Dastidar S., Negroni E., Choi S., Khokhar N., Bigot A. (2023). 3D Human Induced Pluripotent Stem Cell-Derived Bioengineered Skeletal Muscles for Tissue, Disease and Therapy Modeling. Nat. Protoc..

[131] Zschüntzsch J., Meyer S., Shahriyari M., Kummer K., Schmidt M., Kummer S., Tiburcy M. (2022). The Evolution of Complex Muscle Cell In Vitro Models to Study Pathomechanisms and Drug Development of Neuromuscular Disease. Cells.

[132] Caron L., Kher D., Lee K.L., McKernan R., Dumevska B., Hidalgo A., Li J., Yang H., Main H., Ferri G. (2016). A Human Pluripotent Stem Cell Model of Facioscapulohumeral Muscular Dystrophy-Affected Skeletal Muscles. Stem Cells Transl. Med..

[133] Laberthonnière C., Novoa-Del-Toro E.-M., Delourme M., Chevalier R., Broucqsault N., Mazaleyrat K., Streichenberger N., Manel V., Bernard R., Salort Campana E. (2022). Facioscapulohumeral Dystrophy Weakened Sarcomeric Contractility Is Mimicked in Induced Pluripotent Stem Cells-Derived Innervated Muscle Fibres. J. Cachexia Sarcopenia Muscle.

[134] Caputo L., Granados A., Lenzi J., Rosa A., Ait-Si-Ali S., Puri P.L., Albini S. (2020). Acute Conversion of Patient-Derived Duchenne Muscular Dystrophy iPSC into Myotubes Reveals Constitutive and Inducible over-Activation of TGFβ-Dependent pro-Fibrotic Signaling. Skelet. Muscle.

[135] Choi I.Y., Lim H.T., Estrellas K., Mula J., Cohen T.V., Zhang Y., Donnelly C.J., Richard J.P., Kim Y.J., Kim H. (2016). Concordant but Varied Phenotypes among Duchenne Muscular Dystrophy Patient-Specific Myoblasts Derived Using a Human iPSC-Based Model. Cell Rep..

[136] Mournetas V., Massouridès E., Dupont J., Kornobis E., Polvèche H., Jarrige M., Dorval A.R.L., Gosselin M.R.F., Manousopoulou A., Garbis S.D. (2021). Myogenesis Modelled by Human Pluripotent Stem Cells: A Multi-omic Study of Duchenne Myopathy Early Onset. J. Cachexia Sarcopenia Muscle.

[137] Uchimura T., Asano T., Nakata T., Hotta A., Sakurai H. (2021). A Muscle Fatigue-like Contractile Decline Was Recapitulated Using Skeletal Myotubes from Duchenne Muscular Dystrophy Patient-Derived iPSCs. Cell Rep. Med..

[138] Bruge C., Geoffroy M., Benabides M., Pellier E., Gicquel E., Dhiab J., Hoch L., Richard I., Nissan X. (2022). Skeletal Muscle Cells Derived from Induced Pluripotent Stem Cells: A Platform for Limb Girdle Muscular Dystrophies. Biomedicines.

[139] Mateos-Aierdi A.J., Dehesa-Etxebeste M., Goicoechea M., Aiastui A., Richaud-Patin Y., Jiménez-Delgado S., Raya A., Naldaiz-Gastesi N., López de Munain A. (2021). Patient-Specific iPSC-Derived Cellular Models of LGMDR1. Stem Cell Res..

[140] Tanaka A., Woltjen K., Miyake K., Hotta A., Ikeya M., Yamamoto T., Nishino T., Shoji E., Sehara-Fujisawa A., Manabe Y. (2013). Efficient and Reproducible Myogenic Differentiation from Human iPS Cells: Prospects for Modeling Miyoshi Myopathy in Vitro. PLoS ONE.

[141] Kawada R., Jonouchi T., Kagita A., Sato M., Hotta A., Sakurai H. (2023). Establishment of Quantitative and Consistent In Vitro Skeletal Muscle Pathological Models of Myotonic Dystrophy Type 1 Using Patient-Derived iPSCs. Sci. Rep..

[142] Mondragon-Gonzalez R., Perlingeiro R.C.R. (2018). Recapitulating Muscle Disease Phenotypes with Myotonic Dystrophy 1 Induced Pluripotent Stem Cells: A Tool for Disease Modeling and Drug Discovery. Dis. Model. Mech..

[143] Steele-Stallard H.B., Pinton L., Sarcar S., Ozdemir T., Maffioletti S.M., Zammit P.S., Tedesco F.S. (2018). Modeling Skeletal Muscle Laminopathies Using Human Induced Pluripotent Stem Cells Carrying Pathogenic LMNA Mutations. Front. Physiol..

[144] van der Wal E., Herrero-Hernandez P., Wan R., Broeders M., In ’t Groen S.L.M., van Gestel T.J.M., van IJcken W.F.J., Cheung T.H., van der Ploeg A.T., Schaaf G.J. (2018). Large-Scale Expansion of Human iPSC-Derived Skeletal Muscle Cells for Disease Modeling and Cell-Based Therapeutic Strategies. Stem Cell Rep..

[145] Yoshida T., Awaya T., Jonouchi T., Kimura R., Kimura S., Era T., Heike T., Sakurai H. (2017). A Skeletal Muscle Model of Infantile-Onset Pompe Disease with Patient-Specific iPS Cells. Sci. Rep..

[146] Ortuño-Costela M.D.C., Cerrada V., Moreno-Izquierdo A., García-Consuegra I., Laberthonnière C., Delourme M., Garesse R., Arenas J., Fuster García C., García García G. (2022). Generation of the First Human In Vitro Model for McArdle Disease Based on iPSC Technology. Int. J. Mol. Sci..

[147] Yasuno T., Osafune K., Sakurai H., Asaka I., Tanaka A., Yamaguchi S., Yamada K., Hitomi H., Arai S., Kurose Y. (2014). Functional Analysis of iPSC-Derived Myocytes from a Patient with Carnitine Palmitoyltransferase II Deficiency. Biochem. Biophys. Res. Commun..

[148] Gartz M., Haberman M., Sutton J., Slick R.A., Luttrell S.M., Mack D.L., Lawlor M.W. (2023). ACTA1 H40Y Mutant iPSC-Derived Skeletal Myocytes Display Mitochondrial Defects in an In Vitro Model of Nemaline Myopathy. Exp. Cell Res..

[149] Shahriyari M., Islam M.R., Sakib S.M., Rinn M., Rika A., Krüger D., Kaurani L., Gisa V., Winterhoff M., Anandakumar H. (2022). Engineered Skeletal Muscle Recapitulates Human Muscle Development, Regeneration and Dystrophy. J. Cachexia Sarcopenia Muscle.

[150] Afshar Bakooshli M., Lippmann E.S., Mulcahy B., Iyer N., Nguyen C.T., Tung K., Stewart B.A., van den Dorpel H., Fuehrmann T., Shoichet M. (2019). A 3D Culture Model of Innervated Human Skeletal Muscle Enables Studies of the Adult Neuromuscular Junction. eLife.

[151] Osaki T., Uzel S.G.M., Kamm R.D. (2018). Microphysiological 3D Model of Amyotrophic Lateral Sclerosis (ALS) from Human iPS-Derived Muscle Cells and Optogenetic Motor Neurons. Sci. Adv..

[152] Kim J.H., Kim I., Seol Y.-J., Ko I.K., Yoo J.J., Atala A., Lee S.J. (2020). Neural Cell Integration into 3D Bioprinted Skeletal Muscle Constructs Accelerates Restoration of Muscle Function. Nat. Commun..

[153] de Jongh R., Spijkers X.M., Pasteuning-Vuhman S., Vulto P., Pasterkamp R.J. (2021). Neuromuscular Junction-on-a-Chip: ALS Disease Modeling and Read-out Development in Microfluidic Devices. J. Neurochem..

[154] Jiang Y., Torun T., Maffioletti S.M., Serio A., Tedesco F.S. (2022). Bioengineering Human Skeletal Muscle Models: Recent Advances, Current Challenges and Future Perspectives. Exp. Cell Res..

[155] Leng Y., Li X., Zheng F., Liu H., Wang C., Wang X., Liao Y., Liu J., Meng K., Yu J. (2023). Advances in In Vitro Models of Neuromuscular Junction: Focusing on Organ-on-a-Chip, Organoids, and Biohybrid Robotics. Adv. Mater. Deerfield Beach Fla..

[156] Hall B.K., Hall B.K. (2015). Chapter 1—Vertebrate Skeletal Tissues. Bones and Cartilage.

[157] Dalle Carbonare L., Innamorati G., Valenti M.T. (2012). Transcription Factor Runx2 and Its Application to Bone Tissue Engineering. Stem Cell Rev. Rep..

[158] Huang J., Zhang L., Lu A., Liang C. (2023). Organoids as Innovative Models for Bone and Joint Diseases. Cells.

[159] Bartosh T.J., Ylöstalo J.H., Mohammadipoor A., Bazhanov N., Coble K., Claypool K., Lee R.H., Choi H., Prockop D.J. (2010). Aggregation of Human Mesenchymal Stromal Cells (MSCs) into 3D Spheroids Enhances Their Antiinflammatory Properties. Proc. Natl. Acad. Sci. USA.

[160] Kaushik G., Ponnusamy M.P., Batra S.K. (2018). Concise Review: Current Status of Three-Dimensional Organoids as Preclinical Models. Stem Cells.

[161] Merimi M., El-Majzoub R., Lagneaux L., Moussa Agha D., Bouhtit F., Meuleman N., Fahmi H., Lewalle P., Fayyad-Kazan M., Najar M. (2021). The Therapeutic Potential of Mesenchymal Stromal Cells for Regenerative Medicine: Current Knowledge and Future Understandings. Front. Cell Dev. Biol..

[162] Akiva A., Melke J., Ansari S., Liv N., van der Meijden R., van Erp M., Zhao F., Stout M., Nijhuis W.H., de Heus C. (2021). An Organoid for Woven Bone. Adv. Funct. Mater..

[163] Kassem M., Ankersen L., Eriksen E.F., Clark B.F., Rattan S.I. (1997). Demonstration of Cellular Aging and Senescence in Serially Passaged Long-Term Cultures of Human Trabecular Osteoblasts. Osteoporos. Int..

[164] Giffin J.L., Gaitor D., Franz-Odendaal T.A. (2019). The Forgotten Skeletogenic Condensations: A Comparison of Early Skeletal Development Amongst Vertebrates. J. Dev. Biol..

[165] Gomes A.R., Fernandes T.G., Vaz S.H., Silva T.P., Bekman E.P., Xapelli S., Duarte S., Ghazvini M., Gribnau J., Muotri A.R. (2020). Modeling Rett Syndrome With Human Patient-Specific Forebrain Organoids. Front. Cell Dev. Biol..

[166] Tam W.L., Freitas Mendes L., Chen X., Lesage R., Van Hoven I., Leysen E., Kerckhofs G., Bosmans K., Chai Y.C., Yamashita A. (2021). Human Pluripotent Stem Cell-Derived Cartilaginous Organoids Promote Scaffold-Free Healing of Critical Size Long Bone Defects. Stem Cell Res. Ther..

[167] Frenz S., Goek I., Buser M., Salewskij K., Fairley S., Conca R., Drexler N., Jonsson G., Thomas M., Mizoguchi Y. (2022). Generation of Human Induced Pluripotent Stem Cell-Derived Bone Marrow Organoids. Blood.

[168] O’Connor S.K., Katz D.B., Oswald S.J., Groneck L., Guilak F. (2021). Formation of Osteochondral Organoids from Murine Induced Pluripotent Stem Cells. Tissue Eng. Part A.

[169] Freisinger P., Ala-Kokko L., LeGuellec D., Franc S., Bouvier R., Ritvaniemi P., Prockop D.J., Bonaventure J. (1994). Mutation in the COL2A1 Gene in a Patient with Hypochondrogenesis. Expression of Mutated COL2A1 Gene Is Accompanied by Expression of Genes for Type I Procollagen in Chondrocytes. J. Biol. Chem..

[170] Liang G., Lian C., Huang D., Gao W., Liang A., Peng Y., Ye W., Wu Z., Su P., Huang D. (2014). Endoplasmic Reticulum Stress-Unfolding Protein Response-Apoptosis Cascade Causes Chondrodysplasia in a Col2a1 p.Gly1170Ser Mutated Mouse Model. PLoS ONE.

[171] Lin S., Li K., Qi L. (2023). Cancer Stem Cells in Brain Tumors: From Origin to Clinical Implications. MedComm.

[172] Miller K., Ostrom Q., Kruchko C., Patil N., Tihan T., Cioffi G., Fuchs H., Waite K., Jemal A., Siegel R. (2021). Brain and Other Central Nervous System Tumor Statistics, 2021. CA Cancer J. Clin..

[173] Andreatta F., Beccaceci G., Fortuna N., Celotti M., De Felice D., Lorenzoni M., Foletto V., Genovesi S., Rubert J., Alaimo A. (2020). The Organoid Era Permits the Development of New Applications to Study Glioblastoma. Cancers.

[174] Ishaq H., Patel B.C. (2023). Retinoblastoma. StatPearls.

[175] Seigel G.M., Vergara M.N., Furey K., Shah D. (2023). A Retinal Organoid Model of Retinoblastoma. Investig. Ophthalmol. Vis. Sci..

[176] Srimongkol A., Laosillapacharoen N., Saengwimol D., Chaitankar V., Rojanaporn D., Thanomchard T., Borwornpinyo S., Hongeng S., Kaewkhaw R. (2023). Sunitinib Efficacy with Minimal Toxicity in Patient-Derived Retinoblastoma Organoids. J. Exp. Clin. Cancer Res..

